# Multimodal CT radiomics and 2.5D deep learning for preoperative grading of clear cell renal cell carcinoma in older patients: a multicenter study

**DOI:** 10.3389/fonc.2026.1982970

**Published:** 2026-10-30

**Authors:** Zhengkun Wang, Zixin Liang, Dingyang Lv, Shenyi Hu, Huiyu Zhou, Jinshuai Li, Xiaodong Bian, Jiacheng Gao, Tiantian Zhao, Bingxin Gong, Ying Qiao, Weibing Shuang

**Affiliations:** 1 First Hospital of Shanxi Medical University, Taiyuan, Shanxi, China; 2 Shanxi Medical University, Taiyuan, Shanxi, China; 3 Shanxi Provincial People’s Hospital, Taiyuan, Shanxi, China; 4 Shanxi Bethune Hospital, Taiyuan, Shanxi, China; 5 Union Hospital, Tongji Medical College, Huazhong University of Science and Technology, Wuhan, Hubei, China

**Keywords:** clear cell renal cell carcinoma, computed tomography, deep learning, multimodal artificial intelligence, radiomics, WHO/ISUP grade

## Abstract

**Introduction:**

To develop and validate a multimodal CT-based model integrating intra-peritumoral radiomics, 2.5D deep learning (DL), and clinical-radiological features for preoperative prediction of World Health Organization/International Society of Urological Pathology (WHO/ISUP) grade in older patients with clear cell renal cell carcinoma (ccRCC).

**Methods:**

This multicenter retrospective study included 1,103 patients older than 60 years with pathologically confirmed ccRCC from four hospitals. Patients were allocated to a training cohort (n=459), an internal validation cohort (n=198), and two independent external test cohorts (test1, n=227; test2, n=219). WHO/ISUP grades I–II were classified as low grade and grades III–IV as high grade. Radiomics features were extracted from corticomedullary-phase CT images using intratumoral and progressively expanded intra-peritumoral regions of 1, 2, and 3 mm. In parallel, 2D and 2.5D DL features were extracted using ImageNet-pretrained ResNet34 networks, with the 2.5D approach integrating axial, coronal, and sagittal tumor views. After feature selection, five machine learning classifiers were evaluated. The optimal radiomics model, DL model, and clinical model were integrated using stacking-based decision-level fusion. Model performance was assessed using receiver operating characteristic analysis, calibration curves, decision curve analysis (DCA), and SHapley Additive exPlanations (SHAP).

**Results:**

The random forest (RF) model based on intra-peritumoral 3-mm radiomics features achieved areas under the receiver operating characteristic curve (AUCs) of 0.754 (95% CI, 0.683–0.825) and 0.725 (95% CI, 0.655–0.794) in the test1 and test2 cohorts, respectively. The RF model based on 2.5D DL features achieved corresponding AUCs of 0.756 (95% CI, 0.682–0.830) and 0.713 (95% CI, 0.643–0.783). The multimodal combined model achieved numerically higher external AUCs than the selected single-modality models, reaching 0.775 (95% CI, 0.705–0.845) in test1 and 0.732 (95% CI, 0.664–0.800) in test2. DCA demonstrated favorable clinical net benefit for the combined model, while SHAP analysis identified the 2.5D DL component as the largest contributor to model predictions.

**Discussion:**

A multimodal CT-based framework integrating intra-peritumoral radiomics, 2.5D DL, and clinical-radiological information enabled noninvasive preoperative prediction of WHO/ISUP grade in older patients with ccRCC and maintained predictive performance across independent external cohorts. This approach may provide complementary imaging and clinical information for preoperative risk stratification and individualized treatment planning.

## 1 Introduction

Renal cell carcinoma (RCC) is a common urological malignancy (1). Its incidence peaks between 60 and 70 years of age (2). Clear cell RCC (ccRCC), the most common histological subtype, demonstrates greater biological aggressiveness with elevated risks of recurrence and metastasis (3). Clinicopathological features and comorbidities have been associated with renal cell carcinoma prognosis (4–6). The World Health Organization/International Society of Urological Pathology (WHO/ISUP) grading system (introduced in 2016) is the most widely used standard currently (7). Higher histological grades usually correlate with poorer clinical outcomes. Therefore, accurate preoperative prediction of tumor grade is crucial for developing personalized treatment strategies. Although histopathology remains the definitive diagnostic criterion for ccRCC grading, percutaneous biopsy serves as the primary technique for preoperative assessment. However, diagnostic accuracy may be compromised by the quality of the sample and sampling errors, potentially leading to grading inaccuracies (8, 9). Furthermore, biopsy specimens only capture localized tumor regions, failing to fully represent intratumoral heterogeneity—a limitation that may result in underestimation of true tumor aggressiveness (10).

Computed tomography (CT) is the most commonly used imaging technique for the assessment of renal cancer, particularly for tumor staging and evaluating the invasiveness of ccRCC (11, 12). However, its diagnostic accuracy is highly dependent on the subjective judgment of radiologists. In recent years, artificial intelligence (AI) has found widespread application in image analysis. AI analysis based on contrast-enhanced CT images has proven to be an effective method for predicting WHO/ISUP grading (13). CT-based modeling has progressively expanded to peritumoral and multiphase representations, but the contribution of each information source requires independent evaluation. The tumor microenvironment, with its various stromal cells and cytokines, plays a key role in the initiation and progression of tumors (14). Deep learning (DL) has also seen some applications in renal cancer grading prediction (15, 16). For instance, Yang et al. constructed a Transformer model based on CT images to predict the Fuhrman grading of ccRCC (15). Wang et al. developed a deep learning model based on the ResNet50 architecture to predict the histological grading of ccRCC, demonstrating promising performance (16). However, most existing models have been constructed using 2D or 3D images. Given that RCC exhibits heterogeneity in three-dimensional space, a pseudo-RGB three-channel image is constructed by using the largest cross-sectional slices of the tumor in the transverse, sagittal, and coronal planes as input to the deep learning architecture. This multi-planar 2.5D representation captures selected orthogonal views using a 2D network backbone, although it does not represent the complete tumor volume.

Recent studies have evaluated arterial-phase three-slice 2.5D DL across five hospitals (17), multiphase ResNet50 fusion (18), and a two-center DenseNet201 radiomics nomogram (19). Other 2026 studies investigated triphasic CT habitat fractions (20) and tumor/peritumoral multiphase radiomics (21). These approaches show that the value of additional imaging information depends on the population and validation design. Evidence remains limited for multimodal grade prediction specifically in older patients.

In this study, we developed and validated a multimodal AI system integrating corticomedullary-phase (CMP)-based intra-peritumoral radiomics, orthogonal-plane 2.5D DL features, and clinical-radiological features for predicting WHO/ISUP grade in patients older than 60 years. Evaluation in an internal validation cohort and two external cohorts provides population-specific evidence for preoperative risk assessment.

## 2 Materials and methods

### 2.1 Patients’ data

This multicenter retrospective study was approved by the institutional ethics committees of allparticipating hospitals, with waiver of informed consent. We retrospectively collected a total of 1,103 ccRCC patients from four medical centers in China. The mean age of the patients was 67.54 ± 5.02 years. “Older patients” were defined as individuals aged >60 years across all four centers. The flowchart for patient screening is shown in **Supplementary Figure 1**. Patients from June 2018 to May 2024 at the First Hospital of Shanxi Medical University (FHSXMU) were randomly divided into the training set (n = 459) and validation set (n = 198) in a 7:3 ratio. The test1 set included 227 patients who were consecutively treated at Shanxi Provincial People’s Hospital (SXPPH, n = 107) and Shanxi Bethune Hospital (SXBH, n = 120) from July 2021 to December 2023. The test2 set included 219 patients who were consecutively treated at Union Hospital, Tongji Medical College, Huazhong University of Science and Technology (WUHC) from July 2021 to December 2023. The inclusion criteria were as follows (1): Patients with a unilateral, solitary tumor. (2) Chronological age >60 years; (3) Patients who underwent urological or abdominal/pelvic contrast-enhanced CT (CECT) before surgery, with complete radiological documentation. (4) Patients with postoperative pathological confirmation of ccRCC. (5) Pathological grading according to the WHO/ISUP classification, with complete grading information available. Exclusion criteria were as follows (patients meeting one or more of the following criteria were excluded): (1) Patients with purely cystic ccRCC. (2) Patients with incomplete clinical data. (3) Patients who received radiotherapy or neoadjuvant therapy before surgery. (4) Patients with significant artifacts in CECT images that compromise image quality and preclude analysis.

### 2.2 Pathological grade of tumor

The postoperative tissue samples were graded I to IV by the pathologists according to the WHO/ISUP grading criteria. For the purpose of this study, grades I and II were categorized as low-grade, while grades III and IV were classified as high-grade. This binary endpoint did not replace the four-tier pathological grading system.

### 2.3 CT examination and image preprocessing

All patients included in the study underwent preoperative CT scans using a multi-slice CT system while in the supine position. Non-contrast, corticomedullary, nephrographic, and excretory-phase images were available for all patients across the four centers. Postcontrast acquisition delays were 30-35, 60-70, and 260–290 seconds for CMP, nephrographic, and excretory phases, respectively. Two radiologists with extensive clinical experience processed and analyzed the CT images, recording the relevant data. Any discrepancies were resolved through discussion and consensus. CMP was selected to characterize early enhancement heterogeneity using a consistent phase-specific input across centers, rather than because other phases were unavailable. CMP images were retrieved from the Picture Archiving and Communication Systems (PACS) in DICOM format for radiomics and DL feature extraction. Other phases and multiphase fusion were not evaluated. The CT images were resampled in Python to isotropic voxels of 1 mm × 1 mm × 1 mm using linear interpolation. The window width was set to 300, and the window level was set to 40.

Two experienced radiologists, blinded to the pathological results, manually delineated the regions of interest (ROIs) on the images using ITK-SNAP 3.8 software. The tumor contour was traced layer by layer to identify and outline the intratumoral ROIs. The CT images, along with the corresponding ROIs, were saved in NIFTI format for subsequent feature extraction. The intra-peritumoral ROIs were generated by automated outward dilation of the tumor mask by 1, 2, and 3 mm (**Figure 1**), including the whole tumor and its surrounding tissues rather than an isolated shell. Distances >3 mm were not investigated. Expanded ROIs were visually inspected and edited slice by slice using the ITK-SNAP paintbrush tool to exclude adjacent organs, muscles, and non-renal vascular structures. Non-tumorous renal tissue, intrarenal vessels, and perirenal or renal sinus fat within the expansion distance were retained. No anatomical boundary clipping restricted the ROIs to the renal parenchyma. The reproducibility of the segmentation protocol was assessed by calculating inter- and intra-observer consistency. Inter-observer consistency was assessed by having both radiologists independently segment tumors from 20 randomly selected cases. Intra-observer consistency was evaluated by having one radiologist repeat the segmentation of the same 20 cases after a one-month interval, thereby minimizing recall bias. The intra-class correlation coefficient (ICC) was calculated to quantify segmentation reproducibility. Only features demonstrating both intra- and inter-observer ICC values ≥ 0.80 were considered reproducible and included in subsequent radiomics analysis.

**Figure 1:**
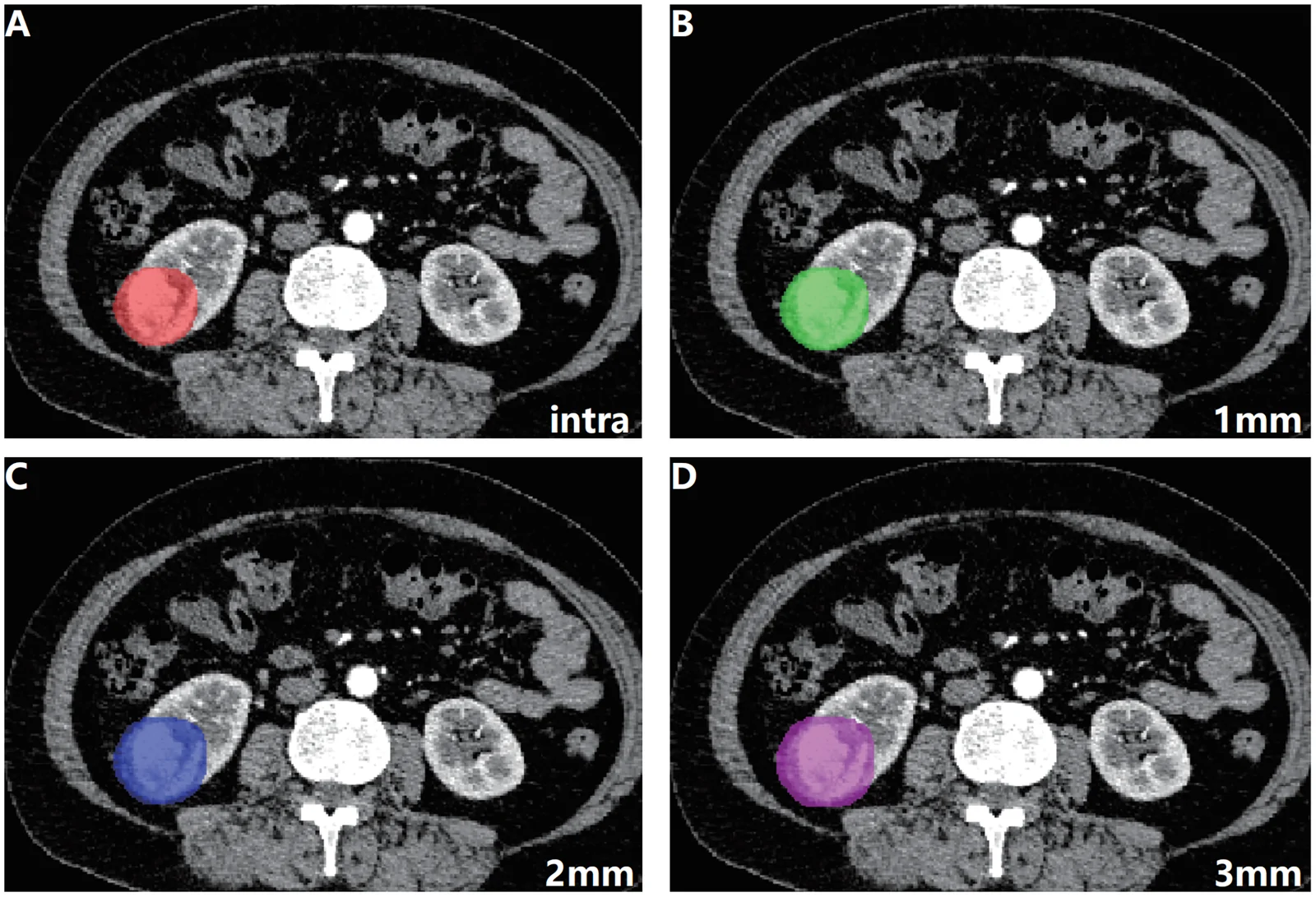
Representative tumor segmentation across intratumoral and intra-peritumoral regions. **(A)** Intratumoral ROI; **(B)** intra-peritumoral 1-mm ROI; **(C)** intra-peritumoral 2-mm ROI; and **(D)** intra-peritumoral 3-mm ROI. ROI, region of interest.

### 2.4 Radiomics feature extraction

Before radiomic feature extraction, the voxel intensities of the corticomedullary-phase CT images were standardized using Z-score normalization. Radiomic features were extracted using PyRadiomics (version 3.0.1) through the OnekeyAI platform (version 3.1.8) in Python 3.7.12 from the intratumoral (Intra), intra-peritumoral-1 mm (Intra-peri-1 mm), intra-peritumoral-2 mm (Intra-peri-2 mm), and intra-peritumoral-3 mm (Intra-peri-3 mm) ROIs, yielding 1,834 features per ROI. These features included 14 shape features, 18 first-order statistical features, 22 grey-level co-occurrence matrix (GLCM) features, 16 grey-level run length matrix (GLRLM) features, 16 grey-level size zone matrix (GLSZM) features, 14 grey-level dependence matrix (GLDM) features, 5 neighboring grey-tone difference matrix (NGTDM) features, and 1,729 derived features. The derived features were transformations based on eight filters: Laplacian of Gaussian (LoG), Wavelet, Local Binary Patterns in 3D (LBP3D), Exponential, Square, SquareRoot, Logarithm, and Gradient. Radiomic feature definitions followed the applicable recommendations of the Image Biomarker Standardisation Initiative (IBSI) (22). Filtered and transformed image features were generated using the corresponding PyRadiomics implementations (23).

### 2.5 Deep learning model development and feature extraction

Following CMP preprocessing (Section 2.3), the 2D input was a tumor patch from the axial slice with the largest tumor cross-sectional area. The 2.5D input combined the largest tumor cross-sections selected separately from the axial, coronal, and sagittal planes. Images were resized to 224 × 224 pixels using linear interpolation, with pixel intensities normalized to zero mean and unit standard deviation. For 2.5D input, the three orthogonal images formed separate channels of a 224 × 224 × 3 pseudo-RGB image.

For each input representation, a two-dimensional ResNet34 network (24) was initialized with ImageNet-pretrained weights and fine-tuned on the training cohort. Both networks used random horizontal and vertical flips and random cropping for augmentation. Training used cross-entropy loss, the Adam optimizer, a learning rate of 0.0001, a batch size of 32, and a maximum of 150 epochs. L2 regularization and ReLU activation were applied, and a softmax classifier provided class probabilities.

After training, 512-dimensional feature vectors were extracted from the global average pooling layer preceding each classification head, generating separate 2D_DL and 2.5D_DL feature sets for feature selection and ML classification (Section 2.6).

### 2.6 Feature selection and single-modality model construction

Given the differential significance of radiomic and deep learning features in discriminating between low- and high-grade ccRCC, we performed independent feature selection for six types of features, including intratumoral radiomics, Intra-peri-1 mm radiomics, Intra-peri-2 mm radiomics, Intra-peri-3 mm radiomics, 2D_DL, and 2.5D_DL features. After extraction, feature values were standardized using Z-score normalization before feature selection. The feature dimensionality reduction and selection process in the training cohort involved multiple analytical stages. First, the Student’s t-test and the Mann-Whitney U test were applied to filter features based on statistical significance (p<0.05). Next, Pearson correlation analysis was conducted to eliminate redundant features, retaining only one representative feature from highly correlated pairs (r>0.9). Subsequently, the mRMR algorithm was employed to select features with maximal relevance to tumor grade and minimal inter-feature redundancy. Finally, LASSO regression with 10-fold cross-validation was performed to identify the most predictive features with nonzero coefficients. The feature selection procedure was applied to the training cohort before classifier cross-validation and was not repeated separately within each classifier-training fold.

After feature dimensionality reduction and selection, the optimized features from the six feature sets were input into support vector machine (SVM), random forest (RF), eXtreme gradient boosting (XGBoost), light gradient boosting machine (LightGBM), and adaptive boosting (AdaBoost) classifiers. Candidate classifiers were assessed using 10-fold cross-validation on the training cohort with the selected features held fixed. Radiomics and DL models were selected based on the evaluated performance metrics.

### 2.7 Clinical model construction

Statistical analysis of clinical and radiological characteristics in the training cohort was performed using both univariate and multivariable logistic regression analyses to determine significant predictors. The identified significant variables were subsequently incorporated into an RF classifier to develop the clinical model.

### 2.8 Multi-modal combined model construction and assessment

Decision-level fusion integrated high-grade probabilities from the Intra-peri-3 mm radiomics RF, 2.5D DL-feature RF, and clinical RF models. The scikit-learn RF meta-learner used n_estimators=201, max_depth=5, min_samples_split=4, and random_state=12; fusion was learned through trees rather than fixed linear coefficients. Base models were fitted on the training cohort, and their predictions for the same patients were used to train the meta-learner. Out-of-fold predictions were not generated, and meta-learner fitting was not performed within cross-validation folds. The fitted ensemble was applied without refitting to the internal validation and external cohorts. Performance, calibration, and clinical utility were assessed as described in Section 2.9; SHapley Additive exPlanations (SHAP) quantified each probability input’s contribution.

### 2.9 Statistical analyses

Statistical analyses were performed using R version 4.3.3 and Python version 3.7.12, with p<0.05 considered statistically significant. Continuous variables were compared using Student’s t-test or the Mann-Whitney U test according to distribution, and ANOVA was used for comparisons across multiple samples. Categorical variables were assessed using the chi-square test. Model performance was evaluated using AUC, accuracy, balanced accuracy, sensitivity, specificity, positive predictive value (PPV), and negative predictive value (NPV), each with a 95% confidence interval. Calibration curves and decision curve analysis (DCA) assessed calibration and clinical utility. **Figure 2** depicts the study workflow.

**Figure 2:**
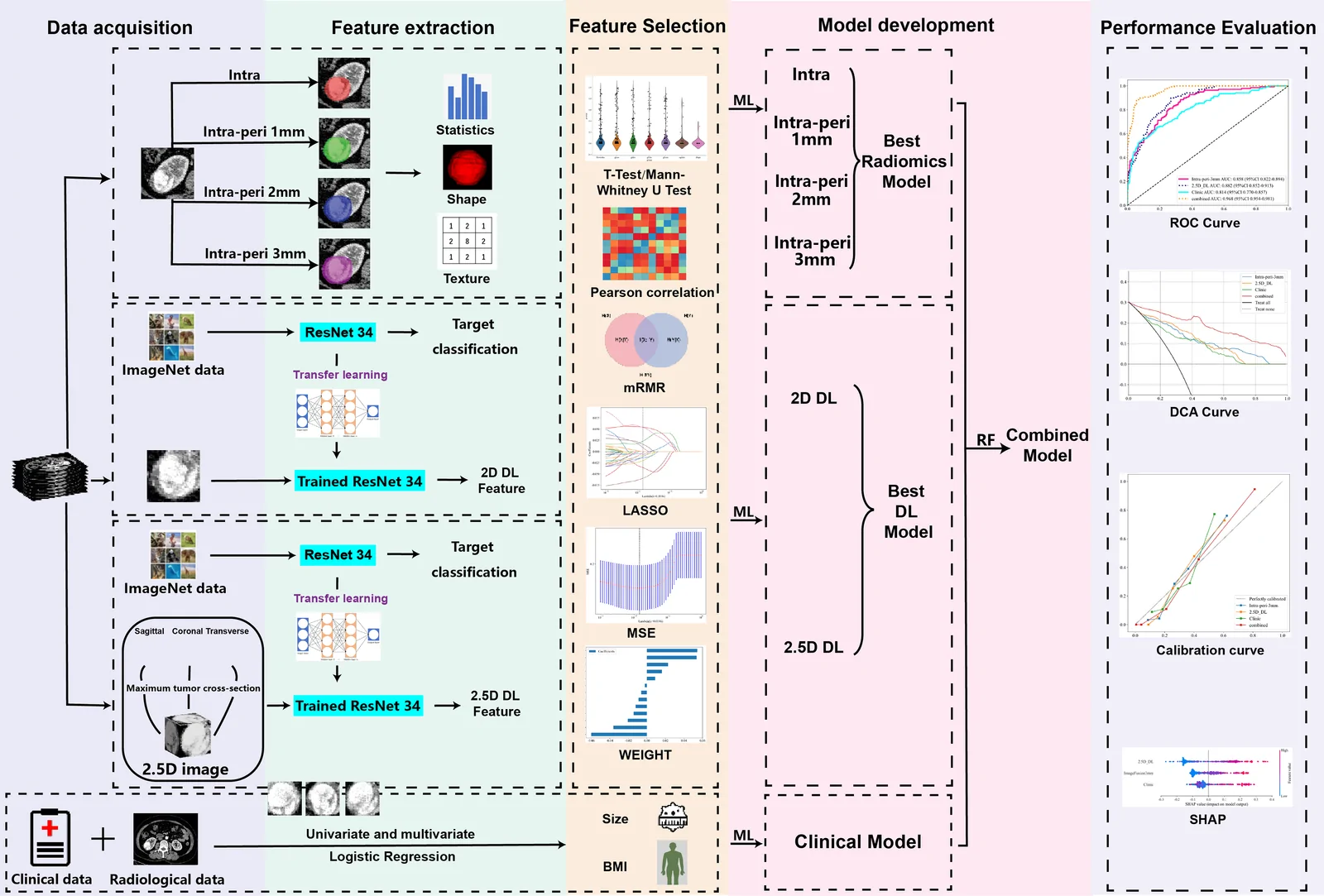
Overall workflow for the development and evaluation of the multimodal prediction model. DL, deep learning; RF, random forest; mRMR, minimum redundancy maximum relevance; ROC, receiver operating characteristic; DCA, decision curve analysis; SHAP, SHapley Additive exPlanations.

Supplementary diagnostic metrics were recalculated from saved patient-level probabilities in Python 3.12.14, with high-grade ccRCC (WHO/ISUP III-IV) as the positive class. Each model’s threshold maximized the training-cohort Youden index and was applied unchanged to validation and external cohorts; probabilities ≥threshold were positive. AUC intervals used the DeLong method; accuracy, sensitivity, specificity, PPV, and NPV intervals used Wilson scores. Balanced accuracy was the mean of sensitivity and specificity. Its 95% interval used the 2.5th and 97.5th percentiles of 5,000 grade-stratified patient bootstrap replicates, with fitted models and thresholds fixed.

## 3 Results

### 3.1 Patient characteristics

The clinical-radiological features of patients are summarized in **Supplementary Table 1**. The baseline data for all datasets were generally balanced, and there were no statistical differences in most clinical-radiological features (P>0.05).

### 3.2 Construction of radiomic models and performance

A total of 1834 radiomic features were extracted respectively from four distinct ROIs. Onlyfeatures with both intra- and interobserver ICCs ≥0.80 were retained for subsequent analysis.After the feature dimensionality reduction and selection process, the Intra, Intra-peri-1 mm, Intra-peri-2 mm and Intra-peri-3 mm retained 9, 18, 13, and 18 optimal radiomic features, respectively. These features were then input into five ML classifiers, resulting in the development of twenty radiomics models. Complete diagnostic metrics, including 95% confidence intervals, for all 20 radiomics models are reported separately by cohort in **Supplementary Tables 2**–**5**. The radar plot in **Figure 3** and **Supplementary Figure 2** illustrates the AUC values of these models. The Intra-peri-3 mm RF was selected as the radiomics component for fusion based on the evaluated performance across both external cohorts. In the test1 and test2 sets, the AUC values of RF classifier for the Intra-peri-3 mm model were 0.754 (95% CI: 0.683 - 0.825) and 0.725 (95% CI: 0.655 - 0.794), respectively.

**Figure 3:**
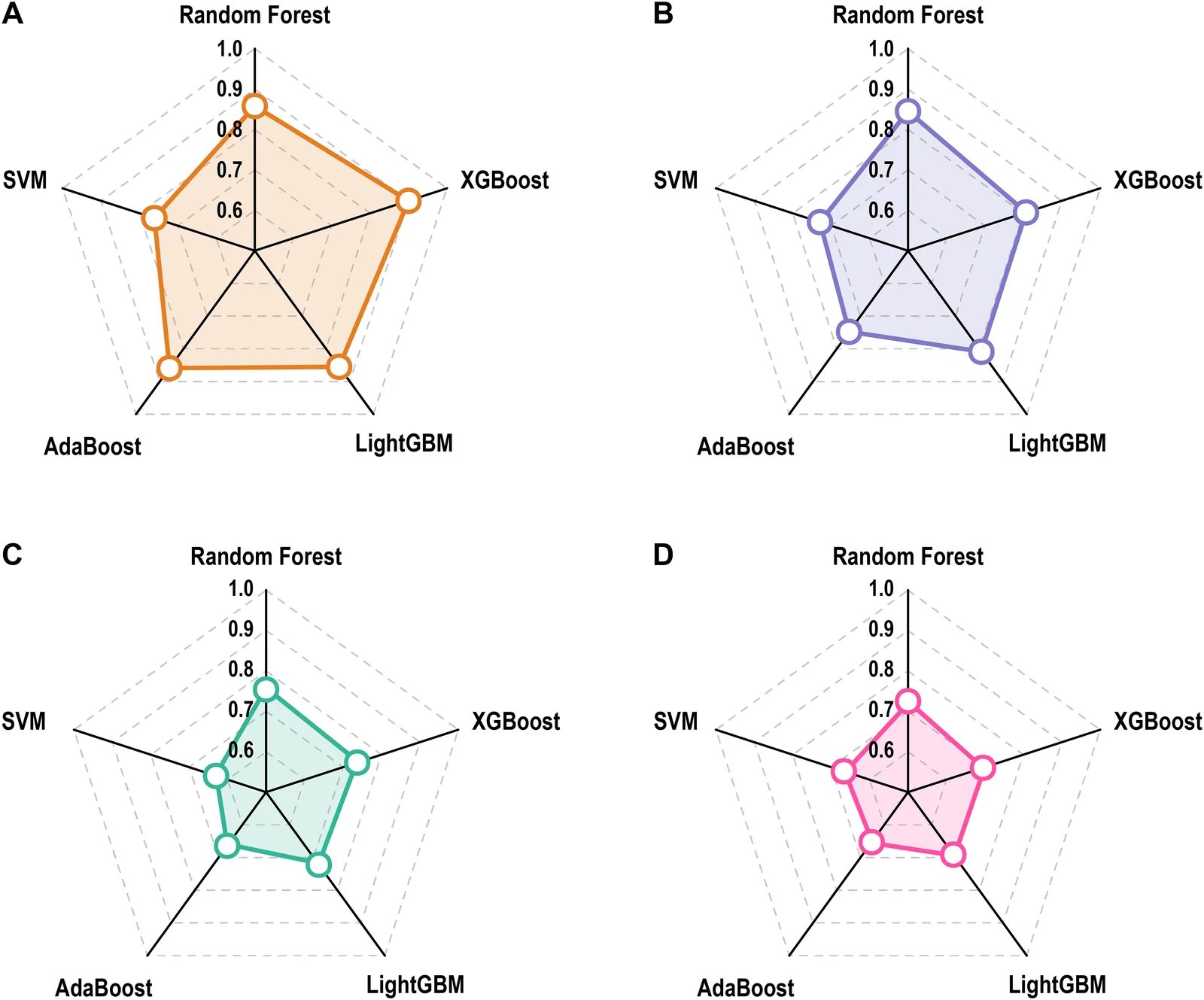
Radar plots comparing the AUCs of five machine-learning classifiers for the intra-peritumoral 3-mm radiomics model. **(A)** Training cohort; **(B)** internal validation cohort; **(C)** external test cohort 1; and **(D)** external test cohort 2. AUC, area under the receiver operating characteristic curve; SVM, support vector machine; RF, random forest; XGBoost, extreme gradient boosting; LightGBM, light gradient boosting machine; AdaBoost, adaptive boosting.

### 3.3 Construction of deep learning models and performance

After training the 2D and 2.5D deep learning models, 512 deep learning features were extractedfrom the penultimate average pooling layer of each model. Then, after the feature dimensionalityreduction and selection process, 22 and 19 optimal DL features were identified, respectively. These features were input into five ML classifiers, resulting in the development of ten DL models. Complete diagnostic metrics, including 95% confidence intervals, for all 10 DL-feature models are reported separately by cohort in **Supplementary Tables 6**, **7**. The petal plot in **Supplementary Figure 3** illustrates the AUC values of these models. The RF classifier for the 2.5D_DL model was selected as the DL-feature component for fusion based on the evaluated performance metrics across the two external cohorts. In the test1 and test2 sets, the AUC values of RF classifier for the 2.5D_DL model were 0.756 (95% CI: 0.682 - 0.830) and 0.713 (95% CI: 0.643 - 0.783), respectively.

### 3.4 Clinical model construction

In the training cohort, univariate and multivariable logistic regression analyses identified tumor size and BMI as independent predictors of pathological grade (**Table 1**). These two predictors were incorporated into an RF classifier to construct the clinicalmodel. The clinical model achieved AUCs of 0.746 (95% CI: 0.678–0.815) and 0.700 (95% CI:0.630–0.769) in external test cohorts 1 and 2, respectively. Complete diagnostic metrics and their 95% confidence intervals for the clinical RF model are reported in **Supplementary Table 8**.

**Table 1:** Univariate and multivariable logistic regression analyses of factors associated with ccRCC grade.

Features	Univariate analysis			Multivariable analysis		
	OR	95% CI	P value	OR	95% CI	P value
BMI	0.968	0.962-0.974	<0.001	0.911	0.869-0.955	0.001
Tumor size	0.945	0.917-0.973	0.002	1.635	1.456-1.837	<0.001
Sex	0.423	0.345-0.520	<0.001	1.05	0.659-1.672	0.864
Marital status	0.446	0.377-0.527	<0.001	0.472	0.121-1.837	0.363
Smoking	0.464	0.345-0.624	<0.001	1.346	0.791-2.293	0.358
Drinking	0.295	0.19-0.459	<0.001	0.567	0.297-1.08	0.147
Hypertension	0.489	0.381-0.627	<0.001	1.163	0.767-1.763	0.551
Diabetes	0.429	0.27-0.681	0.003	1.121	0.608-2.067	0.76
Hematuria	1.08	0.684-1.704	0.782			
Backache	0.493	0.353-0.689	0.001	0.798	0.493-1.29	0.439
Density	0.281	0.219-0.361	<0.001	0.907	0.579-1.42	0.719
Boundary	0.369	0.302-0.452	<0.001	1.029	0.653-1.621	0.918
Enhancement	0.307	0.223-0.422	<0.001	1.134	0.715-1.8	0.653
Involvement of the renal sinus	1.038	0.754-1.429	0.846			

### 3.5 The combined model construction and assessment

The combined model integrated the selected radiomics, 2.5D DL-feature, and clinical outputsthrough an RF meta-learner. Its internal validation AUC was 0.823 (95% CI: 0.760-0.885), comparedwith 0.845, 0.852, and 0.838 for the respective single-modality models. External AUCs were 0.775 (95% CI: 0.705-0.845) and 0.732 (95% CI: 0.664-0.800), numerically higher than those of the selected single-modality models. At the training-derived threshold of 0.415086, external balanced accuracies were 0.756 (95% CI: 0.694-0.816) and 0.678 (95% CI: 0.616-0.736). **Supplementary Table 8** reports complete diagnostic metrics and 95% confidence intervals. **Figure 4** and **Supplementary Figure 4** present metric comparisons and ROC curves; **Figure 5** and **Supplementary Figure 5** present DCA and calibration curves.

**Figure 4:**
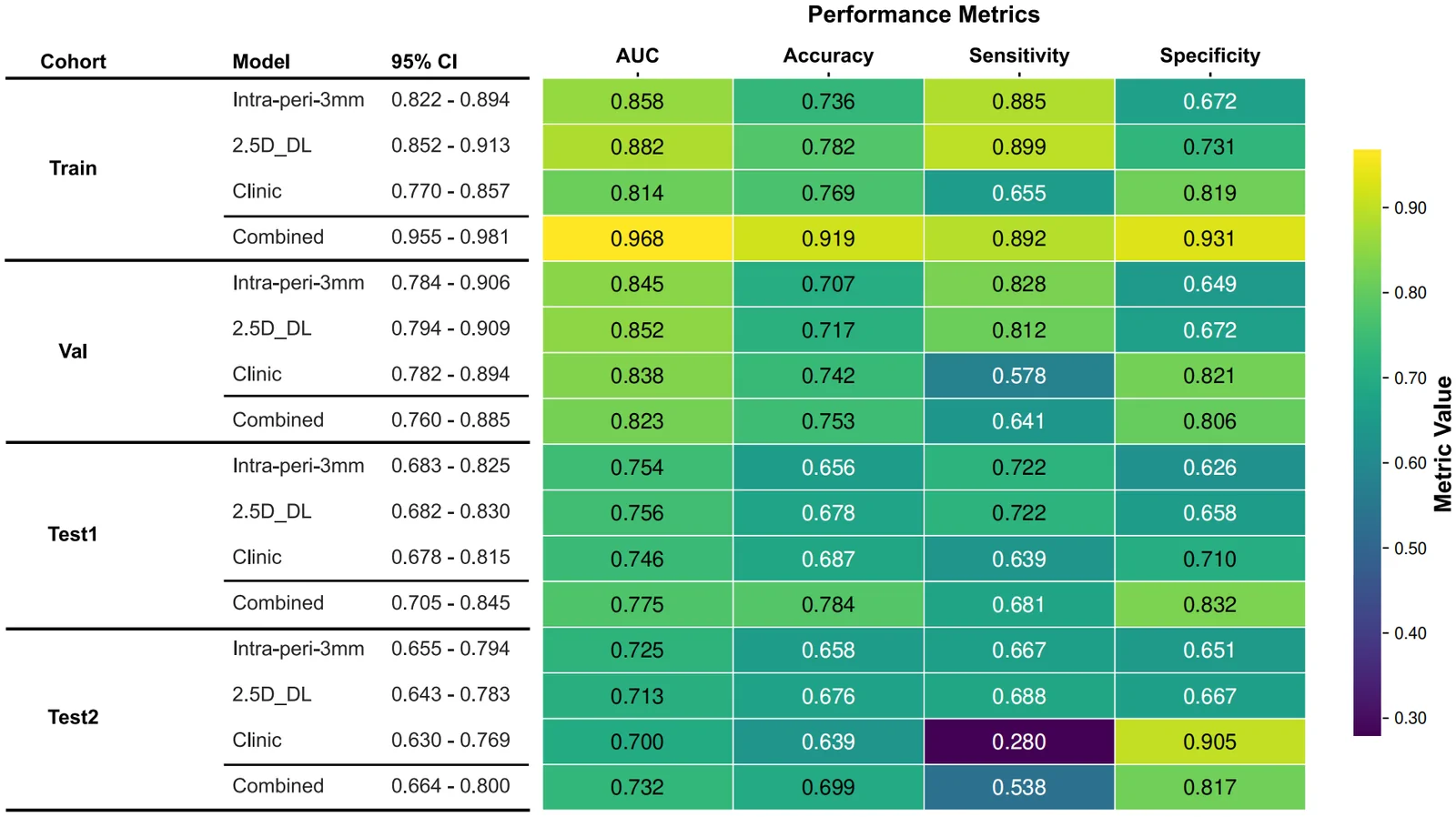
Heatmap comparing the predictive performance of the intra-peritumoral 3-mm radiomics, 2.5D deeplearning, clinical, and combined models across all cohorts. Accuracy, sensitivity, and specificitywere calculated using each model’s training-derived threshold, held fixed across cohorts. Complete diagnostic metrics with 95% confidence intervals, including balanced accuracy, PPV, and NPV, are reported in **Supplementary Tables 2**–**8**. AUC, area under the receiver operating characteristic curve; CI, confidence interval; DL, deep learning.

**Figure 5:**
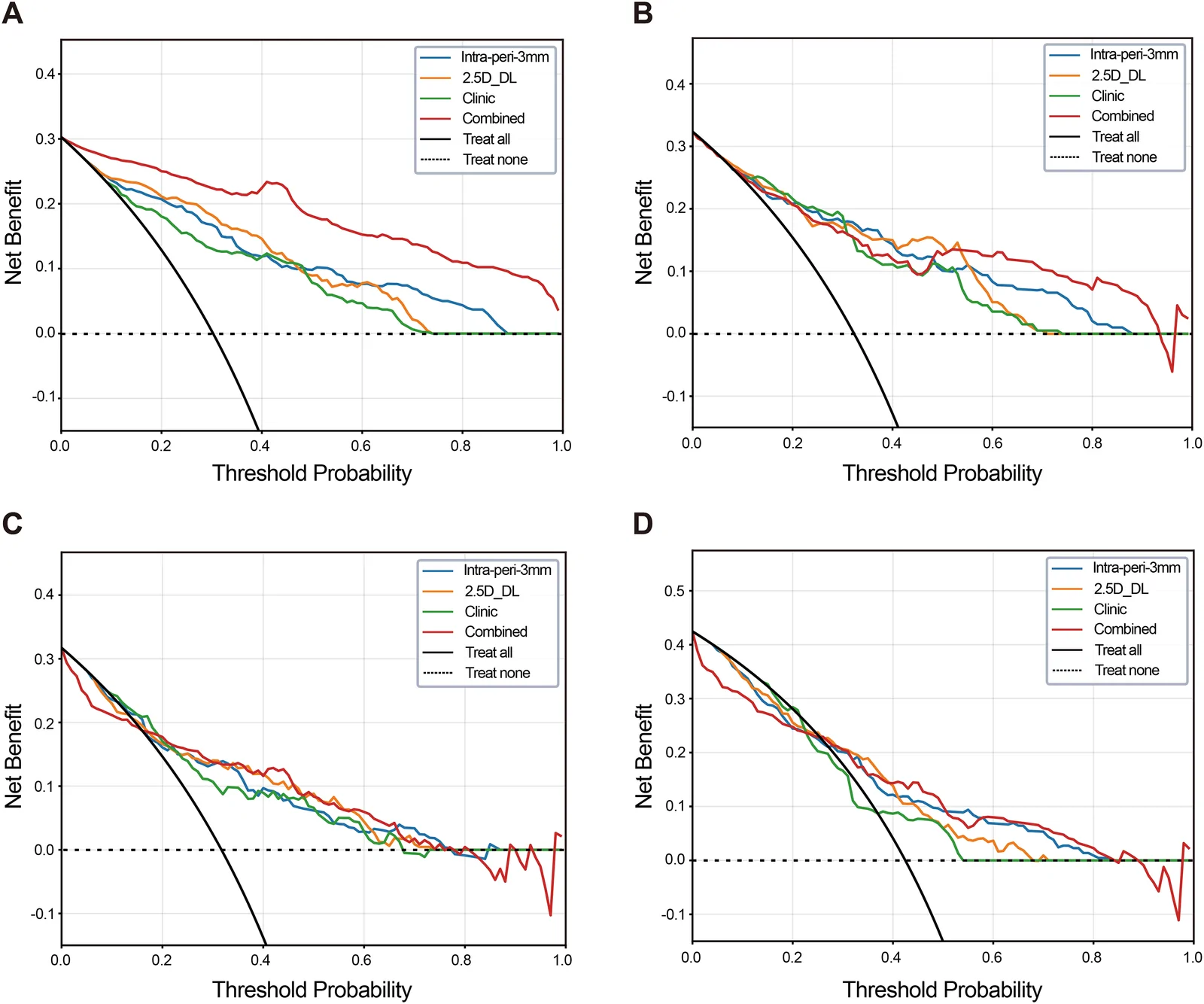
Decision curve analysis of the intra-peritumoral 3-mm radiomics model, 2.5D deep learning model, clinical model, and combined model. **(A)** Training cohort; **(B)** internal validation cohort; **(C)** external test cohort 1; and **(D)** external test cohort 2. The “treat all” and “treat none” strategies are shown as reference curves. DCA, decision curve analysis; DL, deep learning.

### 3.6 Model explanation and visualization

To enhance model interpretability, we generated a SHAP summary plot for the combined model to quantify the contribution of each model component (**Figure 6**). The SHAP summary plot illustrates the distribution and magnitude of the contribution of each component to the model output. The model components were ranked according to their global importance. Each dot represents the SHAP value of a model component for an individual patient, with the horizontal position indicating the direction and magnitude of its contribution to the model output. The color gradient (blue for low values and red for high values) represents the corresponding input value. **Figure 6** shows that the 2.5D_DL component contributed most strongly to the combined model predictions, followed by the ImageFusion3mm and clinical components.

**Figure 6:**
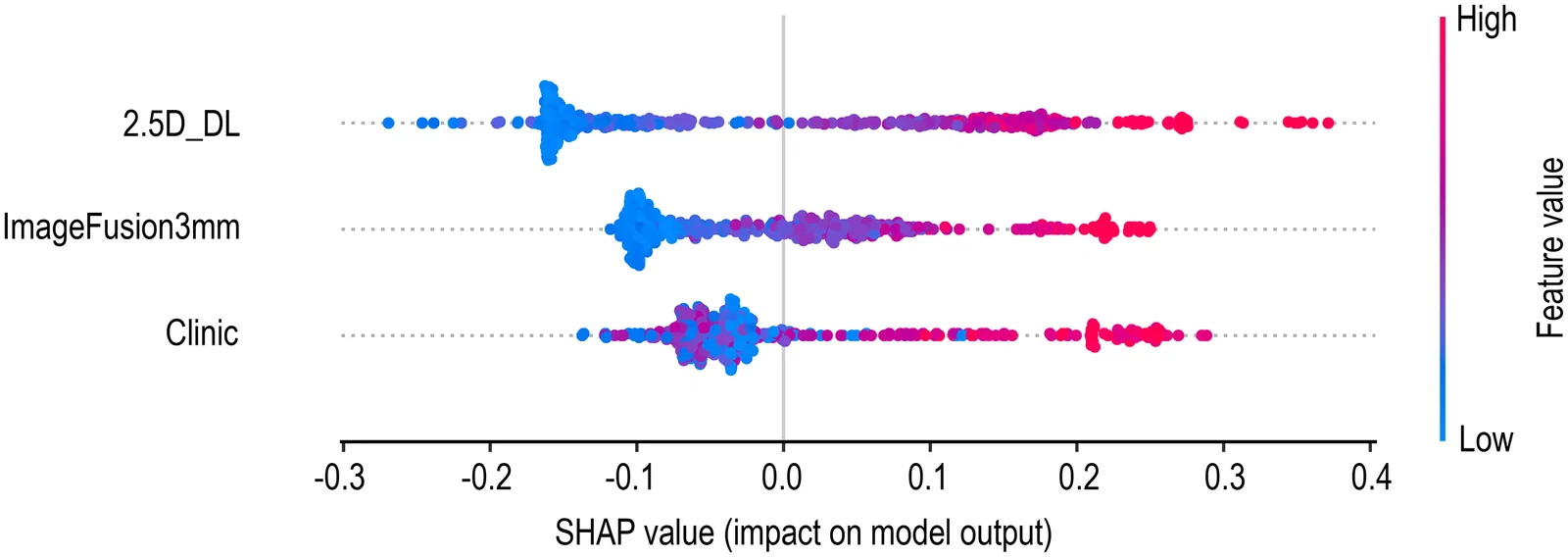
SHAP summary plot illustrating the contribution of each model component to the combined model. 2.5D_DL, 2.5D deep learning model probability; ImageFusion3mm, intra-peritumoral 3-mm radiomics model probability; Clinic, clinical model probability. SHAP, SHapley Additive exPlanations; DL, deep learning.

## 4 Discussion

This study developed and validated an artificial intelligence tool for predicting WHO/ISUP grading in older patients with ccRCC by integrating intratumoral and peritumoral radiomics, 2.5D deep learning features, and clinical-radiological features from contrast-enhanced CT images, utilizing an ensemble learning approach for model fusion. The multimodal combined model achieved external AUCs of 0.775 (95% CI: 0.705-0.845) and 0.732 (95% CI: 0.664-0.800), respectively. All cohorts included patients aged >60 years; applicability to younger or age-unrestricted populations remains untested.

**Supplementary Table 9** compares our outcomes with recent grading studies. Published external AUCs were 0.849-0.870 for three-slice 2.5D DL (17), 0.911 for a multiphase DL-radiomics nomogram (19), and 0.781 (95% CI: 0.599-0.962) for habitat imaging (20). Sun et al. reported an internal test AUC of 0.849 (18), while Muellner et al. reported a cross-validation AUC of 0.697 (95% CI: 0.628-0.765) (21). The table includes reported intervals and within-study statistical comparisons. Differences in age, imaging inputs, cohort size, and validation design limit direct ranking. Our contribution is multicenter evaluation of CMP-based intra-peritumoral and orthogonal-plane fusion in older patients, with two external cohorts.

Accumulating evidence demonstrates that CT- or MRI-based radiomic features can effectively characterize tumor heterogeneity and show correlations with WHO/ISUP grading (13, 25). Demirjian et al. (26) developed a multiphase CT-based radiomics model, with the robust-feature model achieving an AUC of 0.73 for grade classification. Similarly, Xv et al. (27) developed a model combining nephrographic-phase radiomics and clinicoradiological features, achieving an AUC of 0.828 in the testing cohort. Peritumoral tissues may harbor crucial biological information regarding early infiltration, immune microenvironment alterations, and vascular invasion (28, 29). Previous investigations have confirmed the incremental value of peritumoral radiomic features in predicting prognosis of advanced esophageal carcinoma and lymphovascular invasion of hypopharyngeal squamous cell carcinoma (30, 31). This study implemented automated expansion of intratumoral ROIs to incorporate both intratumoral and peritumoral (1 mm/2 mm/3 mm) features. After feature selection, the selected radiomics model (Intra-peri-3 mm with RF classifier) achieved AUCs of 0.754 (95% CI: 0.683-0.825) and 0.725 (95% CI: 0.655-0.794), respectively, showing numerically higher AUCs than models relying solely on intratumoral features. This finding aligns with the work of Demirjian et al. (26), whose comparative analysis similarly identified the RF classifier as demonstrating optimal predictive performance among machine learning algorithms. These findings suggest complementary information from immediately surrounding tissues.

The tumor-host interface may reflect local invasion and peritumoral pseudocapsule alterations (32), providing a physiological rationale for local ROI expansion. Technically, wider expansion adds tissue context but can also introduce unrelated structures. Only 1-, 2-, and 3-mm dilations were tested; 3 mm was the best-performing distance among these options, rather than a universal optimum. Adjacent organs, muscles, and non-renal vessels were manually excluded. Retained renal tissue, intrarenal vessels, and perirenal or renal sinus fat were not separated into individual compartments, and the ROIs were not clipped to renal parenchyma. Their varying tissue composition may influence feature values.

Although all four CT phases were available, CMP provided a common input across centers and limited phase-specific processing. Multiphase features may add baseline attenuation and complementary enhancement information, but require additional segmentation or registration and increase feature redundancy and sensitivity to acquisition differences. Additional phases do not invariably improve grading: Muellner et al. reported AUCs of 0.697 for both nephrographic and multiphase models (21), while Lu et al. favored nephrographic-phase models (33). Our study did not compare phases; therefore, the external results do not establish CMP as optimal or more generalizable than multiphase modeling.

Compared to radiomics relying on handcrafted features, DL can automatically extract latent image features that complement tumor characterization. In the field of RCC grading, Bai et al. (34) developed a predictive model using the RepVGG architecture based on contrast-enhanced ultrasound, achieving an AUC of 0.852 in the test set. Similarly, Yang et al. (15) reported promising predictive performance using a 2D DL model based on arterial-phase CT images. To capture additional spatial information, Wang et al. (16) proposed a 3D-ResNet50 architecture for Fuhrman grade prediction. We selected ImageNet-pretrained ResNet34 (24) for fine-tuning in the 459-patient training cohort. Encoding axial, coronal, and sagittal tumor views as three channels reused a 2D backbone while adding orthogonal spatial context. The 224 × 224 × 3 input avoids dense volumetric feature maps, offering a practical computational compromise. Efficiency gains and superiority over native 3D networks were not benchmarked.

Slice thickness and spacing variations affect through-plane information and can challenge 3D convolutions (35). Isotropic resampling standardized voxel spacing but could not restore missing acquisition detail; reformatted views remained sensitive to native resolution. Our three selected planes also omitted some volumetric heterogeneity. The developed 2.5D DL model achieved AUCs of 0.756 (95% CI: 0.682-0.830) and 0.713 (95% CI: 0.643-0.783) in the two independent test cohorts, respectively, showing numerically higher AUCs than the corresponding 2D DL models. These findings are consistent with Lin et al. (36), who developed a CT-based DL model for ccRCC grading and achieved AUCs of 0.82 and 0.81 in the internal and external test sets, respectively. Although external testing using the TCGA-KIRC cohort was performed, the institutional cohort was derived from a single center and the external test sample was relatively small, warranting further multicenter validation.

Binary grouping was chosen to focus on high-grade disease while pooling the relatively uncommon WHO/ISUP grades I and IV (37). Limited representation of these grades can hinder reliable four-class training and validation. Pathological interobserver variability provides another consideration (38): disagreements within I-II or III-IV do not change the binary label, although II-III disagreement remains consequential. We did not measure grading agreement or compare four-class models. The binary endpoint provides a broad estimate of aggressiveness but does not capture within-group prognostic differences or directly determine partial versus radical nephrectomy.

Our multivariable regression analysis identified tumor size and BMI as independent predictors of ccRCC pathological grade. The association between tumor size and tumor grade is consistent with previous evidence. Wu et al. (39) identified maximal tumor diameter as an independent predictor of high-grade ccRCC. Notably, although these conventional clinical-radiological features provided predictive information for tumor grading, their discriminatory performance was lower than that of the radiomics and deep learning models in the present study. This finding further supports the potential value of multimodal integration for improving preoperative grade prediction.

The model may support preoperative counseling by adding information about tumor aggressiveness. Surgical planning must also consider stage, anatomy, renal function, comorbidities, and patient preferences (40); predicted grade alone should not determine the operation or adjuvant treatment. Other fusion approaches, including Kundu et al.’s CNN framework (41) and Zhu et al.’s multimodal ultrasound network (42), also support combining complementary inputs. Our evaluation extends this approach to CMP radiomics, orthogonal-plane DL, and clinical predictors in an older multicenter population. Prospective studies are needed to establish benefit for clinical decisions and patient outcomes.

Several limitations warrant consideration. The retrospective, age-restricted design requires prospective validation and testing in broader populations. Binary grading, 1–3 mm dilations, CMP-only modeling, and selected 2.5D planes limit the information assessed. Feature selection preceded classifier cross-validation, and the RF meta-learner used in-sample base-model predictions; this may increase overfitting, and classifier-level cross-validation does not evaluate the complete pipeline. Future development should use fold-specific selection and out-of-fold stacking. The model does not localize grade-specific regions and did not incorporate whole-slide pathology images. Finally, literature comparisons describe differing study settings and cannot establish between-study statistical superiority.

## 5 Conclusion

This multicenter study developed an RF-based decision-level fusion model combining 3-mm intra-peritumoral radiomics, triplanar ResNet34 features, and clinical predictors (tumor size and BMI) for preoperative low-/high-grade WHO/ISUP classification in patients older than 60 years. The combined model achieved external AUCs of 0.775 (95% CI: 0.705-0.845) and 0.732 (95% CI: 0.664-0.800), with the 2.5D DL component contributing most to its predictions. These findings support the potential of complementary CT and clinical information for preoperative risk assessment in older patients. Prospective validation is required before clinical implementation.

## Data Availability

The original contributions presented in the study are included in the article/**Supplementary Material**. Further inquiries can be directed to the corresponding authors.
