# Supplementary material for: Multimodal CT radiomics and 2.5D deep learning for preoperative grading of clear cell renal cell carcinoma in older patients: a multicenter study
Source: Front Oncol. 2026 Oct 30;16:1982970. doi: 10.3389/fonc.2026.1982970 (PMC13759392; doi:10.3389/fonc.2026.1982970)
Supplement: Supplementary file 1 [file SupplementaryFile1.docx]

**Supplementary Figure 1 Flowchart for selecting the study population.**

**Supplementary Figure 2** **The petal plot of AUC.** A, B, C, D represent the AUC values of different machine learning classifiers in the training set, validation set, test1 set, and test2 set of the Intratumoral model. E, F, G, H represent the AUC values of different machine learning classifiers in the training set, validation set, test1 set, and test2 set of the Intra-peri-1mm model. I, J, K, L represent the AUC values of different machine learning classifiers in the training set, validation set, test1 set, and test2 set of the Intra-peri-2mm model.

**Supplementary Figure 3 The petal plot of AUC.** A, B, C, D represent the AUC values of different machine learning classifiers in the training set, validation set, test1 set, and test2 set of the 2D_DL model. E, F, G, H represent the AUC values of different machine learning classifiers in the training set, validation set, test1 set, and test2 set of the 2.5D_DL model.

**Supplementary Figure 4 ROC curves for the Intra-peri-3mm model, the 2.5D_DL model, the clinical model (clinic), and combined model.** A, B, C, D represent the ROC curve of different models in the training set, validation set, test1 set, and test2 set, respectively.

**Supplementary Figure 5 The calibration curves for the Intra-peri-3mm model, the 2.5D_DL model, the clinical model (clinic), and combined model.** A, B, C, D represent the calibration curves of different models in the training set, validation set, test1 set, and test2 set, respectively.

Supplementary Table 1 Clinical-radiological features of patients with ccRCC.

| Features | All (n=1103) | Training set (n=459) | Validation set (n=198) | Test1 set (n=227) | Test2 set (n=219) | *P* value |
| --- | --- | --- | --- | --- | --- | --- |
| BMI | 25.10±3.67 | 25.22±3.81 | 25.08±3.40 | 25.41±3.91 | 24.55±3.26 | 0.068 |
| Size | 4.76±2.48 | 4.71±2.46 | 4.67±2.33 | 4.70±2.40 | 5.01±2.71 | 0.433 |
| Sex |  |  |  |  |  | 0.864 |
| female | 371(33.64) | 153(33.33) | 71(35.86) | 77(33.92) | 70(31.96) |  |
| male | 732(66.36) | 306(66.67) | 127(64.14) | 150(66.08) | 149(68.04) |  |
| Marital |  |  |  |  |  | 0.507 |
| other | 13(1.18) | 8(1.74) | 1(0.51) | 2(0.88) | 2(0.91) |  |
| married | 1090(98.82) | 451(98.26) | 197(99.49) | 225(99.12) | 217(99.09) |  |
| Smoking |  |  |  |  |  | 0.319 |
| no | 763(69.17) | 317(69.06) | 144(72.73) | 147(64.76) | 155(70.78) |  |
| yes | 340(30.83) | 142(30.94) | 54(27.27) | 80(35.24) | 64(29.22) |  |
| Drinking |  |  |  |  |  | 0.147 |
| no | 903(81.87) | 380(82.79) | 166(83.84) | 174(76.65) | 183(83.56) |  |
| yes | 200(18.13) | 79(17.21) | 32(16.16) | 53(23.35) | 36(16.44) |  |
| Hypertension |  |  |  |  |  | 0.593 |
| no | 623(56.48) | 261(56.86) | 106(53.54) | 125(55.07) | 131(59.82) |  |
| yes | 480(43.52) | 198(43.14) | 92(46.46) | 102(44.93) | 88(40.18) |  |
| Diabetes |  |  |  |  |  | 0.867 |
| no | 950(86.13) | 399(86.93) | 171(86.36) | 192(84.58) | 188(85.84) |  |
| yes | 153(13.87) | 60(13.07) | 27(13.64) | 35(15.42) | 31(14.16) |  |
| Hematuria |  |  |  |  |  | 0.994 |
| no | 981(88.94) | 407(88.67) | 177(89.39) | 202(88.99) | 195(89.04) |  |
| yes | 122(11.06) | 52(11.33) | 21(10.61) | 25(11.01) | 24(10.96) |  |
| Backache |  |  |  |  |  | 0.692 |
| no | 856(77.61) | 350(76.25) | 159(80.30) | 175(77.09) | 172(78.54) |  |
| yes | 247(22.39) | 109(23.75) | 39(19.70) | 52(22.91) | 47(21.46) |  |
| Density |  |  |  |  |  | 0.097 |
| non-uniform | 526(47.69) | 204(44.44) | 89(44.95) | 117(51.54) | 116(52.97) |  |
| uniform | 577(52.31) | 255(55.56) | 109(55.05) | 110(48.46) | 103(47.03) |  |
| Boundary |  |  |  |  |  | 0.192 |
| unclear | 275(24.93) | 118(25.71) | 59(29.80) | 50(22.03) | 48(21.92) |  |
| clear | 828(75.07) | 341(74.29) | 139(70.20) | 177(77.97) | 171(78.08) |  |
| Enhancement |  |  |  |  |  | 0.501 |
| non-uniform | 759(68.81) | 310(67.54) | 145(73.23) | 153(67.40) | 151(68.95) |  |
| uniform | 344(31.19) | 149(32.46) | 53(26.77) | 74(32.60) | 68(31.05) |  |
| Involvement of the renal sinus |  |  |  |  |  | 0.913 |
| no | 837(75.88) | 353(76.91) | 148(74.75) | 170(74.89) | 166(75.80) |  |
| yes | 266(24.12) | 106(23.09) | 50(25.25) | 57(25.11) | 53(24.20) |  |

Supplementary Table 2 Diagnostic metrics of Intratumoral radiomics model.

A. AUC and classification metrics with 95% confidence intervals.

| Cohort | ML | AUC (95% CI) | Sensitivity (95% CI) | Specificity (95% CI) | Accuracy (95% CI) |
| --- | --- | --- | --- | --- | --- |
| Train | SVM | 0.762 0.7142-0.8098 | 0.799 0.724-0.857 | 0.594 0.539-0.646 | 0.656 0.611-0.698 |
|  | RF | 0.837 0.7988-0.8756 | 0.770 0.693-0.832 | 0.744 0.693-0.788 | 0.752 0.710-0.789 |
|  | XGB | 0.891 0.8603-0.9225 | 0.741 0.662-0.807 | 0.878 0.838-0.910 | 0.837 0.800-0.868 |
|  | LGBM | 0.825 0.7859-0.8639 | 0.763 0.685-0.826 | 0.716 0.664-0.762 | 0.730 0.687-0.768 |
|  | AdaB | 0.833 0.7952-0.8706 | 0.640 0.558-0.715 | 0.841 0.797-0.877 | 0.780 0.740-0.815 |
| Val | SVM | 0.806 0.7383-0.8732 | 0.781 0.666-0.865 | 0.649 0.565-0.725 | 0.692 0.624-0.752 |
|  | RF | 0.86 0.8037-0.9160 | 0.781 0.666-0.865 | 0.761 0.682-0.825 | 0.768 0.704-0.821 |
|  | XGB | 0.794 0.7283-0.8599 | 0.562 0.441-0.677 | 0.821 0.747-0.877 | 0.737 0.672-0.794 |
|  | LGBM | 0.834 0.7747-0.8940 | 0.719 0.599-0.814 | 0.739 0.659-0.806 | 0.732 0.667-0.789 |
|  | AdaB | 0.783 0.7173-0.8483 | 0.500 0.381-0.619 | 0.739 0.659-0.806 | 0.662 0.593-0.724 |
| Test1 | SVM | 0.685 0.6044-0.7659 | 0.708 0.595-0.801 | 0.606 0.528-0.680 | 0.639 0.574-0.698 |
|  | RF | 0.746 0.6762-0.8166 | 0.667 0.552-0.765 | 0.748 0.675-0.810 | 0.722 0.661-0.777 |
|  | XGB | 0.704 0.6312-0.7774 | 0.444 0.335-0.559 | 0.781 0.709-0.839 | 0.674 0.611-0.732 |
|  | LGBM | 0.74 0.6713-0.8087 | 0.611 0.496-0.715 | 0.716 0.641-0.781 | 0.683 0.620-0.740 |
|  | AdaB | 0.724 0.6554-0.7933 | 0.403 0.297-0.518 | 0.781 0.709-0.839 | 0.661 0.597-0.719 |
| Test2 | SVM | 0.692 0.6204-0.7641 | 0.677 0.577-0.764 | 0.595 0.508-0.677 | 0.630 0.564-0.691 |
|  | RF | 0.71 0.6394-0.7800 | 0.591 0.490-0.686 | 0.738 0.655-0.807 | 0.676 0.611-0.734 |
|  | XGB | 0.705 0.6339-0.7754 | 0.430 0.334-0.532 | 0.865 0.795-0.914 | 0.680 0.616-0.739 |
|  | LGBM | 0.71 0.6411-0.7790 | 0.570 0.468-0.666 | 0.722 0.638-0.793 | 0.658 0.592-0.717 |
|  | AdaB | 0.66 0.5873-0.7324 | 0.344 0.255-0.445 | 0.786 0.706-0.848 | 0.598 0.532-0.661 |

Supplementary Table 2 continued. B. Additional diagnostic metrics and training-derived thresholds.

| Cohort | ML | Decision threshold | Balanced accuracy (95% CI) | PPV (95% CI) | NPV (95% CI) |
| --- | --- | --- | --- | --- | --- |
| Train | SVM | 0.259977 | 0.696 0.652-0.738 | 0.461 0.399-0.524 | 0.872 0.821-0.910 |
|  | RF | 0.329369 | 0.757 0.714-0.799 | 0.566 0.495-0.635 | 0.881 0.837-0.915 |
|  | XGB | 0.387703 | 0.810 0.770-0.849 | 0.725 0.647-0.792 | 0.886 0.847-0.917 |
|  | LGBM | 0.315078 | 0.739 0.696-0.782 | 0.538 0.468-0.606 | 0.874 0.828-0.909 |
|  | AdaB | 0.492392 | 0.740 0.696-0.784 | 0.636 0.553-0.711 | 0.843 0.799-0.879 |
| Val | SVM | 0.259977 | 0.715 0.650-0.777 | 0.515 0.417-0.612 | 0.861 0.781-0.916 |
|  | RF | 0.329369 | 0.771 0.710-0.830 | 0.610 0.502-0.708 | 0.879 0.808-0.927 |
|  | XGB | 0.387703 | 0.692 0.626-0.761 | 0.600 0.474-0.714 | 0.797 0.722-0.856 |
|  | LGBM | 0.315078 | 0.729 0.663-0.794 | 0.568 0.459-0.670 | 0.846 0.770-0.900 |
|  | AdaB | 0.492392 | 0.619 0.549-0.692 | 0.478 0.363-0.595 | 0.756 0.676-0.821 |
| Test1 | SVM | 0.259977 | 0.657 0.592-0.721 | 0.455 0.366-0.548 | 0.817 0.737-0.877 |
|  | RF | 0.329369 | 0.708 0.643-0.770 | 0.552 0.447-0.652 | 0.829 0.758-0.882 |
|  | XGB | 0.387703 | 0.613 0.546-0.678 | 0.485 0.368-0.603 | 0.752 0.679-0.812 |
|  | LGBM | 0.315078 | 0.664 0.598-0.729 | 0.500 0.398-0.602 | 0.799 0.724-0.857 |
|  | AdaB | 0.492392 | 0.592 0.526-0.657 | 0.460 0.343-0.582 | 0.738 0.666-0.799 |
| Test2 | SVM | 0.259977 | 0.636 0.572-0.701 | 0.553 0.461-0.641 | 0.714 0.622-0.792 |
|  | RF | 0.329369 | 0.665 0.600-0.729 | 0.625 0.521-0.719 | 0.710 0.627-0.781 |
|  | XGB | 0.387703 | 0.648 0.589-0.706 | 0.702 0.573-0.805 | 0.673 0.597-0.740 |
|  | LGBM | 0.315078 | 0.646 0.583-0.710 | 0.602 0.498-0.698 | 0.695 0.611-0.767 |
|  | AdaB | 0.492392 | 0.565 0.506-0.625 | 0.542 0.417-0.663 | 0.619 0.542-0.690 |

Supplementary Table 3 Diagnostic metrics of Intra-peri-1mm radiomics model.

A. AUC and classification metrics with 95% confidence intervals.

| Cohort | ML | AUC (95% CI) | Sensitivity (95% CI) | Specificity (95% CI) | Accuracy (95% CI) |
| --- | --- | --- | --- | --- | --- |
| Train | SVM | 0.796 0.7533-0.8393 | 0.835 0.764-0.887 | 0.606 0.552-0.658 | 0.675 0.631-0.717 |
|  | RF | 0.837 0.7983-0.8763 | 0.863 0.796-0.911 | 0.656 0.603-0.706 | 0.719 0.676-0.758 |
|  | XGB | 0.897 0.8646-0.9302 | 0.914 0.855-0.950 | 0.750 0.700-0.794 | 0.800 0.761-0.834 |
|  | LGBM | 0.849 0.8126-0.8857 | 0.712 0.632-0.781 | 0.809 0.763-0.849 | 0.780 0.740-0.815 |
|  | AdaB | 0.84 0.8044-0.8765 | 0.763 0.685-0.826 | 0.747 0.697-0.791 | 0.752 0.710-0.789 |
| Val | SVM | 0.821 0.7572-0.8853 | 0.797 0.683-0.877 | 0.649 0.565-0.725 | 0.697 0.630-0.757 |
|  | RF | 0.841 0.7797-0.9015 | 0.875 0.772-0.935 | 0.530 0.446-0.612 | 0.641 0.573-0.705 |
|  | XGB | 0.771 0.6979-0.8443 | 0.766 0.649-0.853 | 0.567 0.483-0.648 | 0.631 0.562-0.695 |
|  | LGBM | 0.809 0.7461-0.8717 | 0.609 0.487-0.719 | 0.784 0.706-0.845 | 0.727 0.661-0.785 |
|  | AdaB | 0.787 0.7175-0.8563 | 0.641 0.518-0.747 | 0.776 0.698-0.838 | 0.732 0.667-0.789 |
| Test1 | SVM | 0.739 0.6714-0.8074 | 0.736 0.624-0.824 | 0.555 0.476-0.631 | 0.612 0.548-0.673 |
|  | RF | 0.732 0.6620-0.8014 | 0.778 0.669-0.858 | 0.529 0.451-0.606 | 0.608 0.543-0.669 |
|  | XGB | 0.725 0.6560-0.7936 | 0.681 0.566-0.777 | 0.606 0.528-0.680 | 0.630 0.565-0.690 |
|  | LGBM | 0.696 0.6227-0.7702 | 0.500 0.387-0.613 | 0.735 0.661-0.799 | 0.661 0.597-0.719 |
|  | AdaB | 0.685 0.6154-0.7544 | 0.514 0.401-0.626 | 0.645 0.567-0.716 | 0.604 0.539-0.665 |
| Test2 | SVM | 0.717 0.6474-0.7865 | 0.677 0.577-0.764 | 0.611 0.524-0.692 | 0.639 0.574-0.700 |
|  | RF | 0.716 0.6452-0.7864 | 0.699 0.599-0.783 | 0.651 0.564-0.728 | 0.671 0.607-0.730 |
|  | XGB | 0.692 0.6212-0.7633 | 0.516 0.416-0.615 | 0.746 0.664-0.814 | 0.648 0.583-0.709 |
|  | LGBM | 0.696 0.6258-0.7655 | 0.376 0.285-0.478 | 0.881 0.813-0.927 | 0.667 0.602-0.726 |
|  | AdaB | 0.659 0.5880-0.7299 | 0.452 0.354-0.553 | 0.690 0.605-0.765 | 0.589 0.523-0.652 |

Supplementary Table 3 continued. B. Additional diagnostic metrics and training-derived thresholds.

| Cohort | ML | Decision threshold | Balanced accuracy (95% CI) | PPV (95% CI) | NPV (95% CI) |
| --- | --- | --- | --- | --- | --- |
| Train | SVM | 0.268586 | 0.720 0.681-0.760 | 0.479 0.417-0.542 | 0.894 0.846-0.928 |
|  | RF | 0.277005 | 0.760 0.720-0.797 | 0.522 0.457-0.585 | 0.917 0.874-0.946 |
|  | XGB | 0.321858 | 0.832 0.799-0.865 | 0.614 0.546-0.677 | 0.952 0.919-0.973 |
|  | LGBM | 0.330456 | 0.761 0.717-0.803 | 0.619 0.542-0.690 | 0.866 0.823-0.900 |
|  | AdaB | 0.484150 | 0.755 0.712-0.797 | 0.567 0.495-0.636 | 0.879 0.835-0.912 |
| Val | SVM | 0.268586 | 0.723 0.658-0.785 | 0.520 0.423-0.617 | 0.870 0.790-0.922 |
|  | RF | 0.277005 | 0.702 0.645-0.759 | 0.471 0.383-0.560 | 0.899 0.813-0.948 |
|  | XGB | 0.321858 | 0.666 0.601-0.734 | 0.458 0.367-0.552 | 0.835 0.746-0.897 |
|  | LGBM | 0.330456 | 0.696 0.628-0.766 | 0.574 0.455-0.684 | 0.808 0.732-0.866 |
|  | AdaB | 0.484150 | 0.708 0.639-0.775 | 0.577 0.462-0.685 | 0.819 0.743-0.876 |
| Test1 | SVM | 0.268586 | 0.645 0.580-0.709 | 0.434 0.350-0.523 | 0.819 0.735-0.881 |
|  | RF | 0.277005 | 0.653 0.589-0.715 | 0.434 0.352-0.520 | 0.837 0.751-0.897 |
|  | XGB | 0.321858 | 0.644 0.576-0.708 | 0.445 0.356-0.539 | 0.803 0.722-0.865 |
|  | LGBM | 0.330456 | 0.618 0.548-0.683 | 0.468 0.360-0.578 | 0.760 0.686-0.821 |
|  | AdaB | 0.484150 | 0.580 0.508-0.650 | 0.402 0.308-0.504 | 0.741 0.661-0.807 |
| Test2 | SVM | 0.268586 | 0.644 0.579-0.709 | 0.562 0.470-0.651 | 0.720 0.628-0.796 |
|  | RF | 0.277005 | 0.675 0.611-0.739 | 0.596 0.502-0.684 | 0.745 0.657-0.818 |
|  | XGB | 0.321858 | 0.631 0.569-0.694 | 0.600 0.490-0.700 | 0.676 0.595-0.748 |
|  | LGBM | 0.330456 | 0.629 0.572-0.686 | 0.700 0.562-0.809 | 0.657 0.582-0.724 |
|  | AdaB | 0.484150 | 0.571 0.505-0.636 | 0.519 0.411-0.624 | 0.630 0.547-0.706 |

Supplementary Table 4 Diagnostic metrics of Intra-peri-2mm radiomics model.

A. AUC and classification metrics with 95% confidence intervals.

| Cohort | ML | AUC (95% CI) | Sensitivity (95% CI) | Specificity (95% CI) | Accuracy (95% CI) |
| --- | --- | --- | --- | --- | --- |
| Train | SVM | 0.763 0.7157-0.8112 | 0.734 0.655-0.800 | 0.656 0.603-0.706 | 0.680 0.636-0.721 |
|  | RF | 0.837 0.7977-0.8765 | 0.741 0.662-0.807 | 0.772 0.723-0.814 | 0.763 0.722-0.799 |
|  | XGB | 0.881 0.8489-0.9134 | 0.806 0.732-0.863 | 0.781 0.733-0.823 | 0.789 0.749-0.824 |
|  | LGBM | 0.839 0.8004-0.8776 | 0.691 0.610-0.761 | 0.819 0.773-0.857 | 0.780 0.740-0.815 |
|  | AdaB | 0.817 0.7779-0.8558 | 0.662 0.580-0.735 | 0.775 0.726-0.817 | 0.741 0.699-0.779 |
| Val | SVM | 0.823 0.7572-0.8883 | 0.797 0.683-0.877 | 0.716 0.635-0.786 | 0.742 0.677-0.798 |
|  | RF | 0.845 0.7852-0.9039 | 0.719 0.599-0.814 | 0.799 0.723-0.858 | 0.773 0.709-0.826 |
|  | XGB | 0.804 0.7378-0.8709 | 0.703 0.582-0.801 | 0.746 0.666-0.812 | 0.732 0.667-0.789 |
|  | LGBM | 0.823 0.7604-0.8847 | 0.703 0.582-0.801 | 0.731 0.651-0.799 | 0.722 0.656-0.780 |
|  | AdaB | 0.759 0.6886-0.8301 | 0.562 0.441-0.677 | 0.769 0.690-0.832 | 0.702 0.635-0.761 |
| Test1 | SVM | 0.722 0.6488-0.7946 | 0.694 0.580-0.789 | 0.645 0.567-0.716 | 0.661 0.597-0.719 |
|  | RF | 0.733 0.6610-0.8056 | 0.625 0.510-0.728 | 0.761 0.688-0.822 | 0.718 0.656-0.773 |
|  | XGB | 0.714 0.6404-0.7872 | 0.569 0.454-0.677 | 0.735 0.661-0.799 | 0.683 0.620-0.740 |
|  | LGBM | 0.71 0.6367-0.7832 | 0.514 0.401-0.626 | 0.742 0.668-0.804 | 0.670 0.606-0.728 |
|  | AdaB | 0.64 0.5628-0.7177 | 0.375 0.272-0.490 | 0.768 0.695-0.827 | 0.643 0.579-0.703 |
| Test2 | SVM | 0.699 0.6286-0.7698 | 0.591 0.490-0.686 | 0.675 0.589-0.750 | 0.639 0.574-0.700 |
|  | RF | 0.706 0.6362-0.7764 | 0.538 0.437-0.635 | 0.770 0.689-0.835 | 0.671 0.607-0.730 |
|  | XGB | 0.69 0.6182-0.7620 | 0.505 0.406-0.605 | 0.698 0.613-0.772 | 0.616 0.551-0.678 |
|  | LGBM | 0.686 0.6125-0.7600 | 0.559 0.458-0.656 | 0.746 0.664-0.814 | 0.667 0.602-0.726 |
|  | AdaB | 0.635 0.5593-0.7099 | 0.366 0.275-0.467 | 0.849 0.776-0.901 | 0.644 0.578-0.704 |

Supplementary Table 4 continued. B. Additional diagnostic metrics and training-derived thresholds.

| Cohort | ML | Decision threshold | Balanced accuracy (95% CI) | PPV (95% CI) | NPV (95% CI) |
| --- | --- | --- | --- | --- | --- |
| Train | SVM | 0.281025 | 0.695 0.650-0.740 | 0.481 0.415-0.548 | 0.850 0.800-0.889 |
|  | RF | 0.319938 | 0.756 0.713-0.798 | 0.585 0.511-0.655 | 0.873 0.829-0.907 |
|  | XGB | 0.339304 | 0.794 0.753-0.832 | 0.615 0.543-0.683 | 0.903 0.862-0.932 |
|  | LGBM | 0.318805 | 0.755 0.711-0.799 | 0.623 0.545-0.696 | 0.859 0.815-0.894 |
|  | AdaB | 0.480974 | 0.718 0.673-0.763 | 0.561 0.484-0.635 | 0.841 0.795-0.878 |
| Val | SVM | 0.281025 | 0.757 0.695-0.818 | 0.573 0.469-0.671 | 0.881 0.807-0.929 |
|  | RF | 0.319938 | 0.759 0.694-0.821 | 0.630 0.515-0.732 | 0.856 0.784-0.907 |
|  | XGB | 0.339304 | 0.725 0.659-0.790 | 0.570 0.460-0.673 | 0.840 0.764-0.895 |
|  | LGBM | 0.318805 | 0.717 0.651-0.783 | 0.556 0.447-0.659 | 0.838 0.760-0.894 |
|  | AdaB | 0.480974 | 0.666 0.596-0.736 | 0.537 0.419-0.651 | 0.786 0.708-0.848 |
| Test1 | SVM | 0.281025 | 0.670 0.604-0.734 | 0.476 0.383-0.571 | 0.820 0.742-0.878 |
|  | RF | 0.319938 | 0.693 0.627-0.755 | 0.549 0.441-0.652 | 0.814 0.743-0.869 |
|  | XGB | 0.339304 | 0.652 0.584-0.718 | 0.500 0.394-0.606 | 0.786 0.713-0.845 |
|  | LGBM | 0.318805 | 0.628 0.559-0.695 | 0.481 0.373-0.590 | 0.767 0.693-0.827 |
|  | AdaB | 0.480974 | 0.571 0.504-0.639 | 0.429 0.314-0.551 | 0.726 0.653-0.788 |
| Test2 | SVM | 0.281025 | 0.633 0.569-0.698 | 0.573 0.473-0.667 | 0.691 0.605-0.766 |
|  | RF | 0.319938 | 0.654 0.591-0.716 | 0.633 0.523-0.731 | 0.693 0.612-0.763 |
|  | XGB | 0.339304 | 0.602 0.539-0.665 | 0.553 0.447-0.654 | 0.657 0.573-0.732 |
|  | LGBM | 0.318805 | 0.653 0.589-0.717 | 0.619 0.512-0.716 | 0.696 0.614-0.768 |
|  | AdaB | 0.480974 | 0.607 0.550-0.664 | 0.642 0.507-0.757 | 0.645 0.569-0.713 |

Supplementary Table 5 Diagnostic metrics of Intra-peri-3mm radiomics model.

A. AUC and classification metrics with 95% confidence intervals.

| Cohort | ML | AUC (95% CI) | Sensitivity (95% CI) | Specificity (95% CI) | Accuracy (95% CI) |
| --- | --- | --- | --- | --- | --- |
| Train | SVM | 0.761 0.7124-0.8089 | 0.640 0.558-0.715 | 0.750 0.700-0.794 | 0.717 0.674-0.756 |
|  | RF | 0.858 0.8221-0.8936 | 0.885 0.821-0.928 | 0.672 0.619-0.721 | 0.736 0.694-0.775 |
|  | XGB | 0.9 0.8711-0.9292 | 0.820 0.748-0.875 | 0.834 0.790-0.871 | 0.830 0.793-0.862 |
|  | LGBM | 0.855 0.8203-0.8903 | 0.784 0.709-0.844 | 0.759 0.710-0.803 | 0.767 0.726-0.803 |
|  | AdaB | 0.859 0.8240-0.8932 | 0.712 0.632-0.781 | 0.828 0.783-0.866 | 0.793 0.754-0.828 |
| Val | SVM | 0.729 0.6546-0.8032 | 0.641 0.518-0.747 | 0.724 0.643-0.793 | 0.697 0.630-0.757 |
|  | RF | 0.845 0.7837-0.9059 | 0.828 0.718-0.901 | 0.649 0.565-0.725 | 0.707 0.640-0.766 |
|  | XGB | 0.807 0.7416-0.8722 | 0.688 0.566-0.788 | 0.799 0.723-0.858 | 0.763 0.699-0.817 |
|  | LGBM | 0.809 0.7453-0.8718 | 0.719 0.599-0.814 | 0.724 0.643-0.793 | 0.722 0.656-0.780 |
|  | AdaB | 0.748 0.6788-0.8168 | 0.453 0.337-0.574 | 0.791 0.715-0.851 | 0.682 0.614-0.743 |
| Test1 | SVM | 0.63 0.5509-0.7082 | 0.528 0.414-0.639 | 0.671 0.594-0.740 | 0.626 0.561-0.686 |
|  | RF | 0.754 0.6827-0.8251 | 0.722 0.610-0.812 | 0.626 0.547-0.698 | 0.656 0.592-0.715 |
|  | XGB | 0.737 0.6652-0.8090 | 0.611 0.496-0.715 | 0.755 0.681-0.816 | 0.709 0.647-0.764 |
|  | LGBM | 0.722 0.6506-0.7935 | 0.611 0.496-0.715 | 0.716 0.641-0.781 | 0.683 0.620-0.740 |
|  | AdaB | 0.664 0.5900-0.7384 | 0.361 0.260-0.476 | 0.813 0.744-0.866 | 0.670 0.606-0.728 |
| Test2 | SVM | 0.667 0.5938-0.7394 | 0.484 0.385-0.584 | 0.754 0.672-0.821 | 0.639 0.574-0.700 |
|  | RF | 0.725 0.6553-0.7940 | 0.667 0.566-0.754 | 0.651 0.564-0.728 | 0.658 0.592-0.717 |
|  | XGB | 0.695 0.6213-0.7689 | 0.516 0.416-0.615 | 0.778 0.698-0.842 | 0.667 0.602-0.726 |
|  | LGBM | 0.691 0.6202-0.7620 | 0.527 0.426-0.625 | 0.738 0.655-0.807 | 0.648 0.583-0.709 |
|  | AdaB | 0.653 0.5791-0.7265 | 0.484 0.385-0.584 | 0.706 0.622-0.779 | 0.612 0.546-0.674 |

Supplementary Table 5 continued. B. Additional diagnostic metrics and training-derived thresholds.

| Cohort | ML | Decision threshold | Balanced accuracy (95% CI) | PPV (95% CI) | NPV (95% CI) |
| --- | --- | --- | --- | --- | --- |
| Train | SVM | 0.363380 | 0.695 0.648-0.741 | 0.527 0.452-0.600 | 0.828 0.780-0.867 |
|  | RF | 0.268567 | 0.778 0.740-0.815 | 0.539 0.475-0.603 | 0.931 0.890-0.957 |
|  | XGB | 0.395013 | 0.827 0.788-0.865 | 0.683 0.609-0.748 | 0.914 0.877-0.941 |
|  | LGBM | 0.321704 | 0.772 0.729-0.814 | 0.586 0.514-0.654 | 0.890 0.847-0.922 |
|  | AdaB | 0.490748 | 0.770 0.725-0.813 | 0.643 0.565-0.714 | 0.869 0.826-0.902 |
| Val | SVM | 0.363380 | 0.682 0.611-0.750 | 0.526 0.416-0.633 | 0.808 0.729-0.869 |
|  | RF | 0.268567 | 0.739 0.678-0.796 | 0.530 0.433-0.625 | 0.888 0.810-0.936 |
|  | XGB | 0.395013 | 0.743 0.677-0.808 | 0.620 0.503-0.724 | 0.843 0.769-0.896 |
|  | LGBM | 0.321704 | 0.721 0.653-0.786 | 0.554 0.447-0.656 | 0.843 0.766-0.899 |
|  | AdaB | 0.490748 | 0.622 0.553-0.691 | 0.509 0.383-0.634 | 0.752 0.674-0.816 |
| Test1 | SVM | 0.363380 | 0.599 0.529-0.666 | 0.427 0.329-0.531 | 0.754 0.676-0.818 |
|  | RF | 0.268567 | 0.674 0.608-0.737 | 0.473 0.382-0.565 | 0.829 0.751-0.887 |
|  | XGB | 0.395013 | 0.683 0.616-0.747 | 0.537 0.429-0.640 | 0.807 0.735-0.863 |
|  | LGBM | 0.321704 | 0.664 0.597-0.727 | 0.500 0.398-0.602 | 0.799 0.724-0.857 |
|  | AdaB | 0.490748 | 0.587 0.524-0.652 | 0.473 0.347-0.602 | 0.733 0.662-0.793 |
| Test2 | SVM | 0.363380 | 0.619 0.557-0.682 | 0.592 0.480-0.696 | 0.664 0.584-0.737 |
|  | RF | 0.268567 | 0.659 0.596-0.723 | 0.585 0.490-0.674 | 0.726 0.637-0.799 |
|  | XGB | 0.395013 | 0.647 0.586-0.709 | 0.632 0.519-0.731 | 0.685 0.605-0.756 |
|  | LGBM | 0.321704 | 0.632 0.570-0.697 | 0.598 0.489-0.697 | 0.679 0.597-0.751 |
|  | AdaB | 0.490748 | 0.595 0.531-0.659 | 0.549 0.441-0.652 | 0.650 0.567-0.724 |

Supplementary Table 6 Diagnostic metrics of 2D_DL model.

A. AUC and classification metrics with 95% confidence intervals.

| Cohort | ML | AUC (95% CI) | Sensitivity (95% CI) | Specificity (95% CI) | Accuracy (95% CI) |
| --- | --- | --- | --- | --- | --- |
| Train | SVM | 0.999 0.9977-1.0000 | 0.993 0.960-0.999 | 0.994 0.978-0.998 | 0.993 0.981-0.998 |
|  | RF | 1 0.9998-1.0000 | 1.000 0.973-1.000 | 0.994 0.978-0.998 | 0.996 0.984-0.999 |
|  | XGB | 1 0.9996-1.0000 | 1.000 0.973-1.000 | 0.991 0.973-0.997 | 0.993 0.981-0.998 |
|  | LGBM | 0.999 0.9977-1.0000 | 0.986 0.949-0.996 | 0.994 0.978-0.998 | 0.991 0.978-0.997 |
|  | AdaB | 1 1.0000-1.0000 | 1.000 0.973-1.000 | 1.000 0.988-1.000 | 1.000 0.992-1.000 |
| Val | SVM | 0.825 0.7638-0.8852 | 0.719 0.599-0.814 | 0.776 0.698-0.838 | 0.758 0.693-0.812 |
|  | RF | 0.802 0.7334-0.8705 | 0.672 0.550-0.774 | 0.784 0.706-0.845 | 0.747 0.683-0.803 |
|  | XGB | 0.762 0.6842-0.8389 | 0.641 0.518-0.747 | 0.769 0.690-0.832 | 0.727 0.661-0.785 |
|  | LGBM | 0.761 0.6858-0.8356 | 0.594 0.471-0.705 | 0.851 0.781-0.901 | 0.768 0.704-0.821 |
|  | AdaB | 0.795 0.7261-0.8635 | 0.641 0.518-0.747 | 0.813 0.739-0.870 | 0.758 0.693-0.812 |
| Test1 | SVM | 0.678 0.5951-0.7599 | 0.569 0.454-0.677 | 0.716 0.641-0.781 | 0.670 0.606-0.728 |
|  | RF | 0.686 0.6033-0.7691 | 0.583 0.468-0.690 | 0.703 0.627-0.770 | 0.665 0.602-0.723 |
|  | XGB | 0.662 0.5816-0.7421 | 0.569 0.454-0.677 | 0.677 0.600-0.746 | 0.643 0.579-0.703 |
|  | LGBM | 0.656 0.5756-0.7362 | 0.458 0.348-0.573 | 0.826 0.758-0.877 | 0.709 0.647-0.764 |
|  | AdaB | 0.696 0.6164-0.7758 | 0.528 0.414-0.639 | 0.781 0.709-0.839 | 0.700 0.638-0.756 |
| Test2 | SVM | 0.639 0.5636-0.7148 | 0.559 0.458-0.656 | 0.714 0.630-0.786 | 0.648 0.583-0.709 |
|  | RF | 0.637 0.5586-0.7156 | 0.591 0.490-0.686 | 0.714 0.630-0.786 | 0.662 0.597-0.721 |
|  | XGB | 0.634 0.5579-0.7092 | 0.570 0.468-0.666 | 0.706 0.622-0.779 | 0.648 0.583-0.709 |
|  | LGBM | 0.616 0.5389-0.6922 | 0.366 0.275-0.467 | 0.802 0.723-0.862 | 0.616 0.551-0.678 |
|  | AdaB | 0.642 0.5669-0.7180 | 0.484 0.385-0.584 | 0.770 0.689-0.835 | 0.648 0.583-0.709 |

Supplementary Table 6 continued. B. Additional diagnostic metrics and training-derived thresholds.

| Cohort | ML | Decision threshold | Balanced accuracy (95% CI) | PPV (95% CI) | NPV (95% CI) |
| --- | --- | --- | --- | --- | --- |
| Train | SVM | 0.198478 | 0.993 0.984-1.000 | 0.986 0.949-0.996 | 0.997 0.982-0.999 |
|  | RF | 0.285375 | 0.997 0.992-1.000 | 0.986 0.950-0.996 | 1.000 0.988-1.000 |
|  | XGB | 0.268993 | 0.995 0.989-1.000 | 0.979 0.940-0.993 | 1.000 0.988-1.000 |
|  | LGBM | 0.402704 | 0.990 0.977-0.998 | 0.986 0.949-0.996 | 0.994 0.978-0.998 |
|  | AdaB | 0.496703 | 1.000 1.000-1.000 | 1.000 0.973-1.000 | 1.000 0.988-1.000 |
| Val | SVM | 0.198478 | 0.747 0.682-0.812 | 0.605 0.493-0.708 | 0.852 0.779-0.905 |
|  | RF | 0.285375 | 0.728 0.658-0.794 | 0.597 0.482-0.703 | 0.833 0.759-0.888 |
|  | XGB | 0.268993 | 0.705 0.635-0.771 | 0.569 0.454-0.677 | 0.817 0.741-0.875 |
|  | LGBM | 0.402704 | 0.722 0.653-0.789 | 0.655 0.527-0.764 | 0.814 0.742-0.870 |
|  | AdaB | 0.496703 | 0.727 0.658-0.793 | 0.621 0.501-0.729 | 0.826 0.752-0.881 |
| Test1 | SVM | 0.198478 | 0.643 0.575-0.710 | 0.482 0.379-0.587 | 0.782 0.707-0.842 |
|  | RF | 0.285375 | 0.643 0.576-0.712 | 0.477 0.376-0.580 | 0.784 0.709-0.844 |
|  | XGB | 0.268993 | 0.623 0.556-0.692 | 0.451 0.352-0.553 | 0.772 0.695-0.835 |
|  | LGBM | 0.402704 | 0.642 0.577-0.706 | 0.550 0.425-0.669 | 0.766 0.697-0.824 |
|  | AdaB | 0.496703 | 0.654 0.588-0.721 | 0.528 0.414-0.639 | 0.781 0.709-0.839 |
| Test2 | SVM | 0.198478 | 0.637 0.573-0.701 | 0.591 0.486-0.688 | 0.687 0.603-0.760 |
|  | RF | 0.285375 | 0.653 0.590-0.717 | 0.604 0.502-0.699 | 0.703 0.619-0.775 |
|  | XGB | 0.268993 | 0.638 0.574-0.702 | 0.589 0.486-0.685 | 0.690 0.606-0.763 |
|  | LGBM | 0.402704 | 0.584 0.526-0.645 | 0.576 0.449-0.694 | 0.631 0.554-0.702 |
|  | AdaB | 0.496703 | 0.627 0.564-0.689 | 0.608 0.494-0.711 | 0.669 0.589-0.740 |

Supplementary Table 7 Diagnostic metrics of 2.5D_DL model.

A. AUC and classification metrics with 95% confidence intervals.

| Cohort | ML | AUC (95% CI) | Sensitivity (95% CI) | Specificity (95% CI) | Accuracy (95% CI) |
| --- | --- | --- | --- | --- | --- |
| Train | SVM | 0.814 0.7745-0.8530 | 0.871 0.805-0.916 | 0.625 0.571-0.676 | 0.699 0.656-0.739 |
|  | RF | 0.882 0.8524-0.9125 | 0.899 0.838-0.939 | 0.731 0.680-0.777 | 0.782 0.742-0.817 |
|  | XGB | 0.923 0.8992-0.9462 | 0.885 0.821-0.928 | 0.825 0.780-0.863 | 0.843 0.807-0.874 |
|  | LGBM | 0.891 0.8621-0.9199 | 0.863 0.796-0.911 | 0.787 0.739-0.829 | 0.810 0.772-0.844 |
|  | AdaB | 0.859 0.8261-0.8919 | 0.921 0.864-0.955 | 0.669 0.615-0.718 | 0.745 0.703-0.783 |
| Val | SVM | 0.811 0.7463-0.8754 | 0.828 0.718-0.901 | 0.627 0.543-0.704 | 0.692 0.624-0.752 |
|  | RF | 0.852 0.7940-0.9091 | 0.812 0.700-0.889 | 0.672 0.588-0.745 | 0.717 0.651-0.775 |
|  | XGB | 0.813 0.7483-0.8779 | 0.719 0.599-0.814 | 0.731 0.651-0.799 | 0.727 0.661-0.785 |
|  | LGBM | 0.826 0.7637-0.8879 | 0.703 0.582-0.801 | 0.754 0.674-0.819 | 0.737 0.672-0.794 |
|  | AdaB | 0.827 0.7638-0.8899 | 0.828 0.718-0.901 | 0.679 0.596-0.752 | 0.727 0.661-0.785 |
| Test1 | SVM | 0.699 0.6223-0.7756 | 0.653 0.538-0.752 | 0.587 0.508-0.662 | 0.608 0.543-0.669 |
|  | RF | 0.756 0.6822-0.8302 | 0.722 0.610-0.812 | 0.658 0.580-0.728 | 0.678 0.615-0.736 |
|  | XGB | 0.751 0.6786-0.8244 | 0.681 0.566-0.777 | 0.723 0.647-0.787 | 0.709 0.647-0.764 |
|  | LGBM | 0.749 0.6764-0.8223 | 0.667 0.552-0.765 | 0.729 0.654-0.793 | 0.709 0.647-0.764 |
|  | AdaB | 0.717 0.6381-0.7949 | 0.708 0.595-0.801 | 0.626 0.547-0.698 | 0.652 0.588-0.711 |
| Test2 | SVM | 0.725 0.6570-0.7924 | 0.731 0.633-0.811 | 0.603 0.516-0.684 | 0.658 0.592-0.717 |
|  | RF | 0.713 0.6430-0.7829 | 0.688 0.588-0.773 | 0.667 0.581-0.743 | 0.676 0.611-0.734 |
|  | XGB | 0.693 0.6229-0.7636 | 0.591 0.490-0.686 | 0.667 0.581-0.743 | 0.635 0.569-0.696 |
|  | LGBM | 0.688 0.6163-0.7595 | 0.613 0.511-0.706 | 0.706 0.622-0.779 | 0.667 0.602-0.726 |
|  | AdaB | 0.69 0.6196-0.7599 | 0.624 0.522-0.715 | 0.667 0.581-0.743 | 0.648 0.583-0.709 |

Supplementary Table 7 continued. B. Additional diagnostic metrics and training-derived thresholds.

| Cohort | ML | Decision threshold | Balanced accuracy (95% CI) | PPV (95% CI) | NPV (95% CI) |
| --- | --- | --- | --- | --- | --- |
| Train | SVM | 0.347632 | 0.748 0.709-0.785 | 0.502 0.439-0.565 | 0.917 0.873-0.947 |
|  | RF | 0.276542 | 0.815 0.780-0.849 | 0.592 0.525-0.656 | 0.944 0.907-0.966 |
|  | XGB | 0.367496 | 0.855 0.821-0.887 | 0.687 0.616-0.751 | 0.943 0.909-0.965 |
|  | LGBM | 0.321657 | 0.825 0.788-0.860 | 0.638 0.567-0.704 | 0.930 0.893-0.955 |
|  | AdaB | 0.467243 | 0.795 0.759-0.828 | 0.547 0.483-0.610 | 0.951 0.915-0.972 |
| Val | SVM | 0.347632 | 0.727 0.666-0.788 | 0.515 0.419-0.609 | 0.884 0.804-0.934 |
|  | RF | 0.276542 | 0.742 0.678-0.803 | 0.542 0.442-0.638 | 0.882 0.806-0.931 |
|  | XGB | 0.367496 | 0.725 0.656-0.791 | 0.561 0.453-0.663 | 0.845 0.768-0.900 |
|  | LGBM | 0.321657 | 0.728 0.659-0.793 | 0.577 0.466-0.680 | 0.842 0.766-0.896 |
|  | AdaB | 0.467243 | 0.754 0.692-0.814 | 0.552 0.453-0.648 | 0.892 0.817-0.939 |
| Test1 | SVM | 0.347632 | 0.620 0.551-0.686 | 0.423 0.336-0.516 | 0.784 0.701-0.850 |
|  | RF | 0.276542 | 0.690 0.625-0.754 | 0.495 0.401-0.589 | 0.836 0.760-0.891 |
|  | XGB | 0.367496 | 0.702 0.639-0.766 | 0.533 0.431-0.631 | 0.830 0.757-0.884 |
|  | LGBM | 0.321657 | 0.698 0.633-0.764 | 0.533 0.431-0.633 | 0.825 0.753-0.879 |
|  | AdaB | 0.467243 | 0.667 0.602-0.733 | 0.468 0.377-0.561 | 0.822 0.743-0.881 |
| Test2 | SVM | 0.347632 | 0.667 0.604-0.729 | 0.576 0.486-0.662 | 0.752 0.660-0.826 |
|  | RF | 0.276542 | 0.677 0.613-0.739 | 0.604 0.509-0.692 | 0.743 0.656-0.815 |
|  | XGB | 0.367496 | 0.629 0.562-0.694 | 0.567 0.468-0.661 | 0.689 0.602-0.764 |
|  | LGBM | 0.321657 | 0.660 0.595-0.722 | 0.606 0.505-0.699 | 0.712 0.627-0.784 |
|  | AdaB | 0.467243 | 0.645 0.580-0.707 | 0.580 0.482-0.672 | 0.706 0.619-0.780 |

Supplementary Table 8 Diagnostic metrics of the clinical RF and multimodal combined RF models.

A. AUC and classification metrics with 95% confidence intervals.

| Cohort | Model | AUC (95% CI) | Sensitivity (95% CI) | Specificity (95% CI) | Accuracy (95% CI) |
| --- | --- | --- | --- | --- | --- |
| Train | Clinical RF | 0.814 0.7701-0.8572 | 0.655 0.572-0.729 | 0.819 0.773-0.857 | 0.769 0.728-0.805 |
|  | Combined RF | 0.968 0.9545-0.9813 | 0.892 0.830-0.934 | 0.931 0.898-0.954 | 0.919 0.891-0.941 |
| Val | Clinical RF | 0.838 0.7824-0.8936 | 0.578 0.456-0.691 | 0.821 0.747-0.877 | 0.742 0.677-0.798 |
|  | Combined RF | 0.823 0.7602-0.8853 | 0.641 0.518-0.747 | 0.806 0.731-0.864 | 0.753 0.688-0.807 |
| Test1 | Clinical RF | 0.746 0.6779-0.8146 | 0.639 0.524-0.740 | 0.710 0.634-0.775 | 0.687 0.624-0.744 |
|  | Combined RF | 0.775 0.7045-0.8445 | 0.681 0.566-0.777 | 0.832 0.766-0.883 | 0.784 0.726-0.833 |
| Test2 | Clinical RF | 0.700 0.6300-0.7694 | 0.280 0.199-0.378 | 0.905 0.841-0.945 | 0.639 0.574-0.700 |
|  | Combined RF | 0.732 0.6643-0.8003 | 0.538 0.437-0.635 | 0.817 0.741-0.875 | 0.699 0.635-0.756 |

B. Additional diagnostic metrics and training-derived thresholds.

| Cohort | Model | Decision threshold | Balanced accuracy (95% CI) | PPV (95% CI) | NPV (95% CI) |
| --- | --- | --- | --- | --- | --- |
| Train | Clinical RF | 0.364176 | 0.737 0.691-0.780 | 0.611 0.531-0.685 | 0.845 0.801-0.881 |
|  | Combined RF | 0.415086 | 0.912 0.882-0.940 | 0.849 0.782-0.898 | 0.952 0.922-0.971 |
| Val | Clinical RF | 0.364176 | 0.700 0.634-0.766 | 0.607 0.481-0.719 | 0.803 0.728-0.861 |
|  | Combined RF | 0.415086 | 0.723 0.654-0.789 | 0.612 0.492-0.720 | 0.824 0.750-0.880 |
| Test1 | Clinical RF | 0.364176 | 0.674 0.607-0.736 | 0.505 0.405-0.606 | 0.809 0.735-0.866 |
|  | Combined RF | 0.415086 | 0.756 0.694-0.816 | 0.653 0.541-0.751 | 0.849 0.783-0.897 |
| Test2 | Clinical RF | 0.364176 | 0.592 0.540-0.643 | 0.684 0.525-0.809 | 0.630 0.557-0.697 |
|  | Combined RF | 0.415086 | 0.678 0.616-0.736 | 0.685 0.571-0.780 | 0.705 0.627-0.773 |

**Notes for Supplementary Tables 2-8**

High-grade ccRCC (WHO/ISUP grades III-IV) is the positive class; low grade (I-II) is the negative class. Train includes 459 patients (139 high grade, 320 low grade); Val includes 198 (64 high grade, 134 low grade); Test1 includes 227 (72 high grade, 155 low grade); Test2 includes 219 (93 high grade, 126 low grade).

Values in the diagnostic-metric columns show the point estimate on the first line and the 95% confidence interval on the second line. For this supplementary analysis, each model threshold was determined by maximizing the Youden index on training-cohort probabilities and applied unchanged to Val, Test1, and Test2. A high-grade probability greater than or equal to the threshold was classified as positive. Thresholds are displayed to six decimal places; all calculations used the full-precision thresholds.

AUC confidence intervals use the DeLong method. Accuracy, sensitivity, specificity, PPV, and NPV confidence intervals use Wilson score intervals. Balanced accuracy is (sensitivity + specificity)/2; its confidence interval uses the 2.5th and 97.5th percentiles of 5,000 class-stratified patient bootstrap replicates with fixed fitted models and thresholds. Bootstrap seeds are 20261002 (Train), 20261003 (Val), 20261004 (Test1), and 20261005 (Test2). Training results are apparent fitted-model performance rather than cross-validated performance of the complete pipeline.

ML, machine learning classifier; RF, random forest; XGB, XGBoost; LGBM, LightGBM; AdaB, AdaBoost; AUC, area under the receiver operating characteristic curve; CI, confidence interval; PPV, positive predictive value; NPV, negative predictive value.

**Supplementary Table 9 Descriptive comparison with recent CT-based ccRCC grading studies**

| **Study** | **Design and inputs** | **Held-out or cross-validation grading performance** |
| --- | --- | --- |
| Yang Z et al. 2025 (17) | 564 patients; five hospitals. Arterial-phase CT; three-slice 2.5D deep learning. Train 283; internal test 122; external 60/38/61. | External AUC (95% CI): 0.870 (0.8076-0.9334) 0.862 (0.7581-0.9658) 0.849 (0.7766-0.9216) External accuracy: 0.827/0.825/0.839. |
| Sun et al. 2026 (18) | 496 patients; one center. Multiphase ResNet50 features, 5-mm expanded slice ROIs, arterial radiomics and clinical variables. Train 347; internal test 149. | Internal test AUC: 0.849 (clinical-imaging fusion); 0.831 (imaging fusion); 0.634 (clinical). Fusion versus imaging: DeLong P = 0.123. No external test cohort. |
| Yang C et al. 2026 (19) | 1,499 patients; two centers. NC, CMP and NP radiomics; DenseNet201 and clinical data; logistic-regression fusion. Train 929; internal 398; external 172. | External AUC: 0.911 (DLRN). Versus radiomics: P = 0.049; versus DL: P = 0.025; versus DLR: P = 0.192. See note on inconsistent published CI limits. |
| Zhang et al. 2026 (20) | 300 patients; two centers. CMP, NP and EP habitat fractions plus sex and tumor size; logistic-regression nomogram. Train 190; internal 82; external 28. | External AUC (95% CI): 0.781 (0.599-0.962). Versus R.E.N.A.L. score: external DeLong P = 0.330. |
| Muellner et al. 2026 (21) | 250 patients; university-affiliated hospitals. Four-phase CT; tumor and 5-mm peritumoral ROIs; Elastic Net/RF. Pooled predictions from 10-fold stratified CV. | Grade AUC (95% CI): 0.697 (0.628-0.765), tumor-region Elastic Net. Sensitivity: 0.147. Nephrographic versus multiphase grade AUC: 0.697/0.697 (P = 0.994). No independent external test cohort. |
| Present study | 1,103 patients >60 years; four hospitals. CMP 3-mm intra-peritumoral radiomics, orthogonal-plane ResNet34 2.5D features and clinical RF; RF meta-learner. Train 459; Val 198; Test1 227; Test2 219. | External AUC (95% CI): Test1 0.7745 (0.7045-0.8445) Test2 0.7323 (0.6643-0.8003) Balanced accuracy (95% CI): 0.7564 (0.6944-0.8157) 0.6775 (0.6158-0.7364) Complete metrics: Tables 2-8. |

Notes: Validation settings are labeled separately, and published P values refer to within-study comparisons, not tests against our model. Differences in populations, thresholds, CT inputs, and sample sizes limit direct comparison. Yang C et al. reported inconsistent external AUC intervals in the Results (0.890-0.925) and Fig. 3 caption (0.850-0.955); only the consistent AUC of 0.911 is reproduced. Present-study estimates use four decimals here and three decimals in the main text. References 17-21 match the manuscript numbering.

AUC, area under the ROC curve; CI, confidence interval; CMP, corticomedullary phase; CV, cross-validation; DL, deep learning; DLR, deep learning radiomics; DLRN, deep learning radiomics nomogram; EP, excretory phase; NC, non-contrast; NP, nephrographic phase; RF, random forest.

**References for Supplementary Table 9**

17.	Yang Z, Jiang H, Shan S, Wang X, Kou Q, Wang C, et al. 2.5D Deep Learning-Based Prediction of Pathological Grading of Clear Cell Renal Cell Carcinoma Using Contrast-Enhanced CT: A Multicenter Study. Acad Radiol 2025; 32:5907-5916.

18.	Sun C, Chen Q, Gao M, He S, Zhang Z, Zhang W, et al. Multi-channel multiphase CT-based deep learning and radiomics fusion model for noninvasive pathological grading of clear cell renal cell carcinoma. Front Oncol 2026; 15:1710329.

19.	Yang C, Zhang Z, Wu B, Zhao H, Ma T, Luo Y, et al. Multiphase CT-based deep learning radiomics nomogram models for preoperative WHO/ISUP grading of clear cell renal cell carcinoma: a two-center validation study. BMC Med Imaging 2026; 26:194.

20.	Zhang L, Shi N, Chen X, Shang S, Lu S, Li T, et al. A multicenter study on preoperative WHO/ISUP grading of clear cell renal cell carcinoma using triphasic contrast-enhanced CT-based habitat imaging. BMC Med Imaging 2026; 26:174.

21.	Muellner M, Pawan SJ, Lei X, Aron M, Desai M, Gurram N, et al. Multiphase CT-Based Tumor and Peritumoral Radiomics for Characterization of Clear Cell Renal Cell Carcinoma. J Imaging Inform Med 2026. Published online January 5, 2026. doi:10.1007/s10278-025-01797-9.
